# Heart energy signature spectrogram for cardiovascular diagnosis

**DOI:** 10.1186/1475-925X-6-16

**Published:** 2007-05-04

**Authors:** Vladimir Kudriavtsev, Vladimir Polyshchuk, Douglas L Roy

**Affiliations:** 1 Biosignetics Corporation, Toronto, Canada; 2 Biosignetics Corporation, 29 Downing Ct, Exeter, New Hampshire 03833, USA; 3 Department of Cardiology, Izaak Walton Killam Children's Health Center, Dalhousie Medical School, Halifax, Nova Scotia, Canada

## Abstract

A new method and application is proposed to characterize intensity and pitch of human heart sounds and murmurs. Using recorded heart sounds from the library of one of the authors, a visual map of heart sound energy was established. Both normal and abnormal heart sound recordings were studied. Representation is based on Wigner-Ville joint time-frequency transformations. The proposed methodology separates acoustic contributions of cardiac events simultaneously in pitch, time and energy. The resolution accuracy is superior to any other existing spectrogram method. The characteristic energy signature of the innocent heart murmur in a child with the S3 sound is presented. It allows clear detection of S1, S2 and S3 sounds, S2 split, systolic murmur, and intensity of these components. The original signal, heart sound power change with time, time-averaged frequency, energy density spectra and instantaneous variations of power and frequency/pitch with time, are presented. These data allow full quantitative characterization of heart sounds and murmurs. High accuracy in both time and pitch resolution is demonstrated. Resulting visual images have self-referencing quality, whereby individual features and their changes become immediately obvious.

## 1.0 Background

### 1.1 Cardiac auscultation

Cardiac auscultation is a difficult skill to acquire and today most medical students graduate without the ability to determine whether a heart sound or murmur is normal or abnormal [1,2]. Evidence also indicates that this skill is not acquired later in practice [3,4]. There is question that despite improved heart sound teaching methods [5] whether improvement in this clinical skill has occurred. This diagnostic deficit results in, (a) certain patients with an undiagnosed organic cardiac lesion will suffer ill health or possible death at a later date, or (b) in the case of the innocent murmur, present in at least 72% of normal children [6,7], expensive cardiac investigation must be carried out to reach this diagnosis. The availability of a new quantitative digitally based computer method, such as herein described, and which with high accuracy can determine and quantify key heart sound variations (i.e. frequency/pitch, intensity, timing, energy, sound split and ejection click), will present a valuable asset to the delivery of health care.

The approach used is based on spectrograms representing a dynamic graphic image of heart sound intensity in time and frequency. At present, current methods of spectral display are not generally understood or even employed in clinical medicine. We propose new method and format that will enable better characterization of heart sounds and hopefully will present a new foundation for subsequent clinical implementation and testing.

### 1.2 Phonocardiograms (PCG)

The PCG is a display of the heart sound signal showing that heart sounds and murmurs can provide useful information to the physician by complementing cardiac auscultation. Basic methodology of distinguishing cardiac murmurs from the PCG is the same as interpreting murmurs from auscultation. However, it provides additional information about timing of cardiac phases and events as well as serving as a digital record that can be utilized to characterize dynamic changes associated with therapy and course of the disease. PCG complements auscultation.

The major PCG clinical drawback is that it does not present information on frequency (pitch) of heart sounds and their components, one of the major deciding factors for murmur clinical interpretation. It does not have the ability to differentiate separate multiple (folded) frequencies of various sounds and presents no information concerning dynamic changes of energy (power) stored in the sound. Other deficiencies arguably include signal filtration effects (change of visual representation due to filtration) and presence of artifacts and noises that can visually mask weak sounds. Challenges in pinpointing start and end points of certain sounds have been reported. End point positions will also depend on the applied filter, which add additional uncertainty. Manual segmentation (separation of heart sound components) may be another problem as well.

PCG never achieved acceptance as a routine clinical investigative method [8], but did find a valuable place in clinical investigation and research. However current newly developed "system science" [9] and signal processing computational technologies in combination with a digital sound recording technologies, electronic recording stethoscopes, advanced new vibration sensors [10] and finally extraordinary computing power now afforded to PDA's, tablet PCs, palm PCs, laptops and MP3 players/recorders make it now possible to completely revitalize old PCG-based approaches.

### 1.3 Heart sound spectrography

The concept of heart sound spectral display was first introduced by McKusick in 1955 [11] and in a subsequent series of his clinical publications [12-14]. (Victor A. McKusick, M.D., Professor of Medical Genetics and Cardiologist by training, The Johns Hopkins University School of Medicine and a physician-scientist widely acknowledged as the father of genetic medicine, is a recipient of the National Medal of Science, 2002.) This display provides added frequency (pitch) dimension to the PCG signal display. While this work did not receive significant reception by clinicians, there is now a renewed interest in this approach both in clinical medicine and in biomedical engineering research. Using spectrograms obtained by McKusick, Don Michael [15] illustrated the intrinsic properties of various heart lesions in his monograph "Auscultation of the Heart". Similar works has been recently reported by Balster et al. [16], Nopponen and Lukkarinen [18,19]. Tovar-Corona et al. [20,21], Bhatikar et al. [22], Tuchinda and Thompson [26,27] utilized wavelet-based transform to obtain time varying scalogram maps. The spectrogram offers additional insight into time dependent change of murmur frequency. Donnerstein [17] correlated spectrogram frequency characteristics with Doppler echo velocity. Tavel & Katz [23,24] reported a method of clinical differentiation of aortic stenosis from innocent murmur using spectrogram measurements. Finally, Tavel [25] indicated great promise for this approach for clinical diagnosis.

Unfortunately methods presented in [9-19,23-25] use various forms of the Short Term Fast Fourier Transform (STFT) to obtain instantaneous frequency characteristics of signals, and all these methods are subject to the "quantum uncertainty" theorem, which states that a signal and its Fourier transform can not both have small support [32], and that frequency and time and both can not be determined to arbitrary precision [45,47]. The resulting outcome of this drawback is a non-unique, low fidelity image, which changes depending on its frequency resolution [29,30]. Also, heart sounds are nonlinear, non-sinusoidal and exponential signals, and it has been demonstrated in signal processing literature [43] that Fourier transform is not mathematically appropriate method to study such signals.

Tuchinda and Thompson [26], Tovar-Corona et al. [20,21], Bhatikar et al. [22] utilized continuous wavelet based transformation (CWT) to develop spectrogram looking maps that present wavelet scale variation in time (scaleograms). CWT approach is not as well established in clinical studies as traditional spectrogram approach [8-19] and is presently emerging. Unlike for STFT spectrograms, time and frequency resolution of the CWT is non-uniform in the entire time-frequency domain [31]. At high frequencies, there is good time resolution and bad frequency resolution. At low frequencies, we have better frequency resolution and bad time resolution. Accordingly, this results in smearing the time-frequency representation of the signal in time at low frequencies. The speed of wavelet transform computation and improved resolution over the STFT are the primary reasons that the wavelet transforms have become a popular analysis tool [32]. Graphic results presented by Tuchinda and Thompson [26] also fail to provide sufficient qualitative resolution and have a strong visual "skewness" as compared to traditional spectrograms.

There are numerous recent publications on the subject of digital recording and analysis of heart sounds. Green et al. [33] discuss optimal ways of recording heart murmur findings using SNOMED templates, DeGroff et al. [22] suggest a potential for computerized frequency analysis to further improve the accuracy of murmur assessment and Nigam et al. [34,35] introduce new ways of segmenting heart sound signal. Finley et al. [36] demonstrated that email digital recordings of children's heart sounds are of diagnostic quality and allow accurate distinction between innocent and pathologic murmurs in >90% of cases. Marcus et al. [37] and Collins et al. [38] use heart sound recordings to correlate S3 estimates, BNP levels and CHF diagnosis, demonstrating very high specificity (85–90%) of digital heart sound recordings for CHF diagnosis in patients over 50 years old. Kudriavtsev et al. [39] demonstrated that Still's murmurs have narrow spectral bandwidth, with this being a significant feature differentiating them from abnormal murmurs.

We conclude that there is a clear upsurge of clinical interest in spectrographic representation of heart sounds. However existing signal processing methods lack in accuracy and resolution. Unlike other short term Fourier transform based approaches [15-19] and Gabor's transformation [32,48,49], which offer approximation to instantaneous energy distribution of a signal, the Wigner-Ville distribution [45,46] has been derived to compute the signal energy at each time instant, exactly utilizing knowledge of the entire signal to compute time-frequency properties for each moment in time. The method used in this study is based on the Wigner-Ville distribution and is called Heart Energy Signature (HES).

HES represents a unique state of dynamically changing multi-component signal of the heart beat. It can be visualized as an image of the instantaneous heart pulsation energy distribution in both frequency and time domains. It is intended for use as an individual biometric characteristic for heart sound interpretation.

## 2.0 Methods

### 2.1 Wigner-Ville distribution function

The pseudo Wigner Ville Distribution [40-42] is a form of the spectrogram, which is based on joint time-frequency distribution (Eugene Paul Wigner (1902–1995), Nobel prize laureate in Physics, introduced quasi-probability distribution in 1932 study quantum corrections to classical statistical mechanics). Wigner's probability function [44,45] addresses a question of the Heisenberg quantum uncertainty [47] – that momentum and position of the particle can not be determined to arbitrary precision (quantum physics theory). For a quantum particle described by its probability function of coordinates, Wigner has developed a probability distribution of the particle to simultaneously have particular coordinates and momentum [45]. Ville [46] further developed Wigner's function to compute the instantaneous frequency of the signal at each time instant. The resulting Wigner-Ville distribution of time and frequency [32,40,41] attempts to express frequency as a function of time. Since signal frequency is related to signal energy, one can interpret Wigner-Ville distribution as the energy map of a signal in time and frequency.

### 2.2 Heart energy signature (HES)

**Figure 1:**
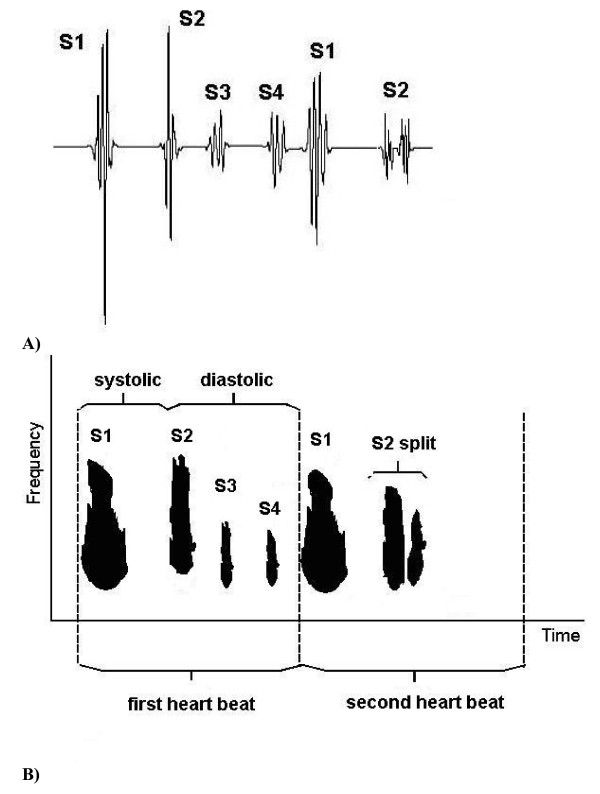
(A, B). Schematics of the Heart Sound Structure and Energy Signature Map. A) Schematic heart sound s wave form display (phonocardiogram -PCG). Second heart beat shows schematically S2 split. B) Energy Signature joint time-frequency map.

**Figure 2:**
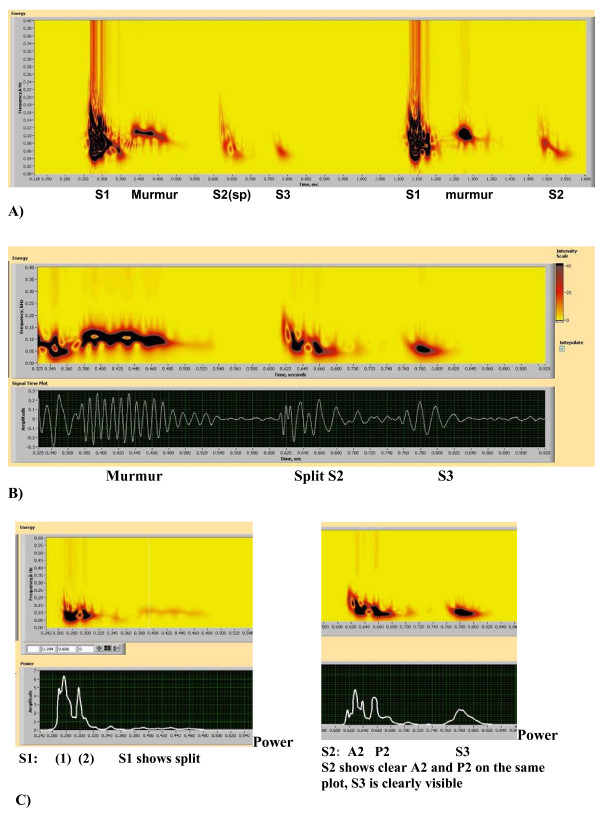
(A, B). Heart Energy Signature Spectrogram obtained using pseudo Wigner-Ville joint time frequency distribution. A) Two consecutive heart beats showing S1 heart sound, innocent murmur, S2 heart sound and S3 heart sound. Period between S1 and S2 is systolic and between the S2 and neighboring S1 diastolic. For display purposes 0.4 sec of diastolic period (between S3 and S1 were cut out of the image [see Additional file 1]. B) "Zoom in" on the first heart beat showing end of S1, murmur, S2 and S3 sounds. C) Image Detail – Same Spectrogram as shown in Figs. 2(A,B).

A Heart Energy Signature is essentially a high-resolution 2D spectrographic image of the heart sound signal that is based on the Wigner-Ville joint time frequency distribution [40] of recorded heart sound signal. Schematic details are shown in Fig. 1, where the corresponding heart sound components (Fig. 1A) and matching elements on the energy signature map (Fig. 1B) are identified. This image stores comprehensive information concerning time averaged and instantaneous changes in mechanical energy of the heart beat. These changes are characterized by frequency and intensity. Unlike previous attempts to characterize heart sounds in this manner (based on Short Term Fourier Transform) STFT (and/or Gabor transform) the HES method is unique in its ability to resolve heart energy accurately in both time and frequency simultaneously. STFT spectrogram can only resolve accurately in time or frequency, but not both time and frequency [28]. Figs. 2(A,B) presents a typical example of a HES obtained from a patient (showing two heart beats) and can be compared with spectrograms obtained using traditional short term window Fourier transform (Figs. 9(A,B), 10(A–D)) and Table 2. This is of a pediatric patient with innocent heart murmur recorded at the apex, sampling frequency of 11 kHz. Binary wave file with the sound is attached [see Additional file 1]. Other comparisons are shown on Figs. 5(A–E), 6(A–E), 7(A–C), 8(A–D) and Table 1. We have thus a method having accurate time-frequency resolution and which satisfies the many mathematical properties, including energy, time and frequency marginals and instantaneous frequency [32,49,53].

**Table 1:** Murmur frequency resolution for STFT (Fig. 4A, and Figs. 7(A-D))

**Window size, w**	**Mean frequency, Hz**	**Half-bandwidth, Hz**	**Peak frequency, Hz**	**Low Frequency, Hz**	**High frequency, Hz**
HES	107.5	53.7	112.9	53.76	161.29
16	114.4	114.4	37.68	0	228.79
256	113.0	24.22	99.59	88.8	137.3
1024	113.0	21.53	102.28	91.5	123.6

Sampling frequency – 11 kHz. Half bandwidth, low frequency and high frequency are estimated from the spectral plot at 50% of maximum magnitude.

**Table 2:** Quantitative comparisons of murmur frequency resolution as presented on Fig. 2B

	**Murmur**		**S1**		**S2**	
	**HES**	**STFT**	**HES**	**STFT**	**HES**	**STFT**

Half-bandwidth HB, Hz	20	101	36	80	28	84
Low frequency LF, Hz	80	0	32	0	48	0
High frequency HF, Hz	120	201	104	161	105	169

Tovar-Corona et al. [20,21] described similarly appearing contour graphs obtained using the continuous wavelet (CWT) based method. This method is now gaining acceptance in signal processing and mathematical details of this method are discussed elsewhere [30-32]. The CWT method presents results in wavelet scale – time map, and not on frequency (pitch) time map. Thus, accurately correlating wavelet scales with frequencies is difficult and for this reason is beyond the scope of this study.

### 2.3 Mathematical method and computational implementation

#### 2.3.1 Preprocessing

The flowchart for computation of Heart Energy Signature (HES) is shown in the Appendix A [see Figure 15]. The first step in pre-processing heart sounds is normalization of the data. By doing so, the heart sounds obtained from different instruments and measurements may be compared. That is, normalization makes data instrument and measurement independent. The amplitude of heart sounds may vary widely, depending upon the location of the sensor used and the measurement system, e.g. phonocardiograph (PCG) vs. electronic stethoscope. To standardize the comparison of heart sounds in the time domain, they are normalized to have their amplitude vary between [-1,+1]. The process of normalization of the signal *x*(*t*) to [-1,+1] amplitude range is well known in signal processing. The basic steps include:

1. find the minimum *x*_min _and the maximum *x*_max _values of the signal

2. divide the signal by 0.5*|*x*_max _- *x*_min_|

Presentation of the time signal in the normalized form is important, since the same signal can appear differently at different amplitude scales. Furthermore, normalization of the heart signal creates the signal presentation with easily computed proportionality relationships between the amplitudes of the signal at various time instances.

After normalization, the next step in the heart sound processing is computation of heart sound energy, as described in the following section.

#### 2.3.3 HES derivation using joint time frequency transformation

The energy of a signal *x*(*t*), including both acoustic and PCG signals, is proportional to the squared amplitude of the signal. The signal energy *E*, contained at the time interval [t, t+T] is computed as

E=∫tt+T|x(t)|2dt
MathType@MTEF@5@5@+=feaafiart1ev1aaatCvAUfKttLearuWrP9MDH5MBPbIqV92AaeXatLxBI9gBaebbnrfifHhDYfgasaacH8akY=wiFfYdH8Gipec8Eeeu0xXdbba9frFj0=OqFfea0dXdd9vqai=hGuQ8kuc9pgc9s8qqaq=dirpe0xb9q8qiLsFr0=vr0=vr0dc8meaabaqaciaacaGaaeqabaqabeGadaaakeaacqWGfbqrcqGH9aqpdaWdXbqaaiabcYha8jabdIha4jabcIcaOiabdsha0jabcMcaPiabcYha8naaCaaaleqabaGaeGOmaidaaOGaemizaqMaemiDaqhaleaacqWG0baDaeaacqWG0baDcqGHRaWkcqWGubava0Gaey4kIipaaaa@41AA@

The time plot of the heart sound PCG displays the amplitude of the sound at each instant, i.e. no information about the energy is displayed. An accepted principle in acoustics is that the energy of the single frequency acoustic signal at each instant is proportional to the squared amplitude of the signal and the squared frequency of the signal. Computation of the acoustic energy is particularly difficult where the acoustic signal consists of many signals with fast changing frequency components. In this case, the acoustic energy must be presented in the form reflecting its energy content at each instant at the various frequencies contained in the signal. Thus, one must compute acoustic energy as a function of both time and frequency: *E *= *E*(*t*, *f*).

The best method to compute heart sound energy is to utilize joint time-frequency distribution (JTFD). JTFD reflects the distribution of the signal energy in the time-frequency plane [51,52]. However, JTFD may not mathematically satisfy energy properties, i.e. to be positive throughout time-frequency plane. In order for distribution to have the same properties as energy, the chosen distribution has been modified to be a real positive value at each point of the time-frequency plane. Steps to obtain such distribution are outlined below.

A large number of time-frequency distributions of a signal *x*(*t*) are given by Cohen's class as

C(t,f)=12π∭φ(θ,τ)x(t+τ2)x*(t−τ2)e−jθt−j2πfτ−jθudτdudθ,
 MathType@MTEF@5@5@+=feaafiart1ev1aaatCvAUfKttLearuWrP9MDH5MBPbIqV92AaeXatLxBI9gBaebbnrfifHhDYfgasaacH8akY=wiFfYdH8Gipec8Eeeu0xXdbba9frFj0=OqFfea0dXdd9vqai=hGuQ8kuc9pgc9s8qqaq=dirpe0xb9q8qiLsFr0=vr0=vr0dc8meaabaqaciaacaGaaeqabaqabeGadaaakeaacqWGdbWqcqGGOaakcqWG0baDcqWGSaalcqWGMbGzcqGGPaqkcqWGGaaicqGH9aqpcqWGGaaidaWcaaqaaiabigdaXaqaaiabikdaYGGaciab=b8aWbaadaWddaqaaiab=z8aMjabcIcaOiab=H7aXjabcYcaSiab=r8a0jabcMcaPaWcbeqab0Gaey4kIiVaey4kIiVaey4kIipakiabdIha4jabcIcaOiabdsha0jabdccaGiabgUcaRiabdccaGmaalaaabaGae8hXdqhabaacbaGae4NmaidaaiabcMcaPiabdccaGmaavacabeqabSqaaiabdQcaQaGcbaGaemiEaGhaaiabcIcaOiabdsha0jabdccaGiabgkHiTiabdccaGmaalaaabaGae8hXdqhabaGae4NmaidaaiabcMcaPiabdccaGmaavacabeqabSqaaiabgkHiTiabdQgaQjab=H7aXjabdsha0jabgkHiTiabdQgaQjab+jdaYiab=b8aWjabdAgaMjab=r8a0jabgkHiTiabdQgaQjab=H7aXjabdwha1bGcbaGaemyzaugaaiabdccaGiabdsgaKjab=r8a0jabdsgaKjabdwha1jabdsgaKjab=H7aXjab+XcaSaaa@7C17@

where t is time, *f *is frequency and *τ *is the running time. The function *φ*(*θ*, *τ*) is the kernel defining distribution properties. If the kernel *φ*(*θ*, *τ*) = 1, we obtain the Wigner-Ville Distribution (WVD):

WVDxx(t,f)=12π∫−∞∞x(t+τ2)x* (t−τ2)e−j2πfτ dτ,
MathType@MTEF@5@5@+=feaafiart1ev1aaatCvAUfKttLearuWrP9MDH5MBPbIqV92AaeXatLxBI9gBaebbnrfifHhDYfgasaacH8akY=wiFfYdH8Gipec8Eeeu0xXdbba9frFj0=OqFfea0dXdd9vqai=hGuQ8kuc9pgc9s8qqaq=dirpe0xb9q8qiLsFr0=vr0=vr0dc8meaabaqaciaacaGaaeqabaqabeGadaaakeaacqWGxbWvcqWGwbGvcqWGebardaWgaaWcbaGaemiEaGNaemiEaGhabeaakiabcIcaOiabdsha0jabdYcaSiabdAgaMjabcMcaPiabdccaGiabg2da9iabdccaGmaalaaabaGaeGymaedabaGaeGOmaidcciGae8hWdahaamaapehabaGaemiEaGNaeiikaGIaemiDaqNaemiiaaIaey4kaSIaemiiaaYaaSaaaeaacqWFepaDaeaaieaacqGFYaGmaaGaeiykaKIaemiiaaYaaubiaeqabeWcbaGaemOkaOcakeaacqWG4baEaaGaeeiiaaIaeiikaGIaemiDaqNaeyOeI0IaemiiaaYaaSaaaeaacqWFepaDaeaacqGFYaGmaaGaeiykaKIaemiiaaYaaubiaeqabeWcbaGaeyOeI0IaemOAaOMae4NmaiJae8hWdaNaemOzayMae8hXdqhakeaacqWGLbqzaaGaeeiiaaIaemizaqMae8hXdqNae4hlaWcaleaacqGHsislcqGHEisPaeaacqGHEisPa0Gaey4kIipaaaa@69ED@

The WVD can be regarded as theoretically optimal in that a maximum number of desirable mathematical properties are satisfied [51]. In the field of the signal processing all time-frequency distributions of Cohen's class can be computed by means of convolution of the Wigner distribution with a two-dimensional impulse response function [52].

For the kernel *φ*(*θ*, *τ*) = *μ*(*τ*), we obtain the pseudo WVD (PWVD). The Gaussian sliding window function *μ*(*τ*) is used having an optimal time-frequency concentration:

PWVDxx(t,f)=12π∫−∞∞x(t+τ2)x*(t−τ2)μ(τ)e−j2πfτdτ,
MathType@MTEF@5@5@+=feaafiart1ev1aaatCvAUfKttLearuWrP9MDH5MBPbIqV92AaeXatLxBI9gBaebbnrfifHhDYfgasaacH8akY=wiFfYdH8Gipec8Eeeu0xXdbba9frFj0=OqFfea0dXdd9vqai=hGuQ8kuc9pgc9s8qqaq=dirpe0xb9q8qiLsFr0=vr0=vr0dc8meaabaqaciaacaGaaeqabaqabeGadaaakeaacqWGqbaucqWGxbWvcqWGwbGvcqWGebardaWgaaWcbaGaemiEaGNaemiEaGhabeaakiabcIcaOiabdsha0jabdYcaSiabdAgaMjabcMcaPiabdccaGiabg2da9iabdccaGmaalaaabaGaeGymaedabaGaeGOmaidcciGae8hWdahaamaapehabaGaemiEaGNaeiikaGIaemiDaqNaey4kaSYaaSaaaeaacqWFepaDaeaaieaacqGFYaGmaaGaeiykaKYaaubiaeqabeWcbaGaemOkaOcakeaacqWG4baEaaGaeiikaGIaemiDaqNaeyOeI0IaemiiaaYaaSaaaeaacqWFepaDaeaacqGFYaGmaaGaeiykaKIae8hVd0MaeiikaGIae8hXdqNaeiykaKYaaubiaeqabeWcbaGaeyOeI0IaemOAaOMae4NmaiJae8hWdaNaemOzayMae8hXdqhakeaacqWGLbqzaaGaemiiaaIaemizaqMae8hXdqNae4hlaWcaleaacqGHsislcqGHEisPaeaacqGHEisPa0Gaey4kIipaaaa@6C50@

μ(τ)=h(τ2)h*(−τ2),
 MathType@MTEF@5@5@+=feaafiart1ev1aaatCvAUfKttLearuWrP9MDH5MBPbIqV92AaeXatLxBI9gBaebbnrfifHhDYfgasaacH8akY=wiFfYdH8Gipec8Eeeu0xXdbba9frFj0=OqFfea0dXdd9vqai=hGuQ8kuc9pgc9s8qqaq=dirpe0xb9q8qiLsFr0=vr0=vr0dc8meaabaqaciaacaGaaeqabaqabeGadaaakeaaiiGacqWF8oqBcqGGOaakcqWFepaDcqGGPaqkcqGH9aqpcqWGObaAcqGGOaakdaWcaaqaaiab=r8a0bqaaiabikdaYaaacqGGPaqkcqWGObaAdaahaaWcbeqaaiabcQcaQaaakiabcIcaOiabgkHiTmaalaaabaGae8hXdqhabaGaeGOmaidaaiabcMcaPiabcYcaSaaa@415B@

*h*(*τ*) = *A *exp(-*σ*^2^*τ*^2^),

where *A *and *σ *are real positive constants.

The WVD and PWVD may not necessary be positive functions at each point on the time-frequency domain for general signals. From the energy concept, it would be more convenient to work with a positive function, as in the case of magnitude of FFT. The WVD can artificially be made positive by simply calculating its absolute value at each point. The common interpretation of WVD as an energy density can thus be allowed, or the intensity of a signal, to be simultaneous in time and frequency.

Since for general signals, the WVD takes on negative values, the absolute positive form |*PWVD*_*xx*_(*t*,*f*)| of the PWVD is used in the format for the HES. This guarantees the distribution to be positive in the time-frequency plane and makes straightforward interpretation of the distribution as the signal energy in the time-frequency.

The absolute positive form of the PWVD is used for computation of the HES. Thus, the preferred method to compute heart sound energy distribution is as follows:

*E*(*t*, *f*) = |*PWVD*_*xx*_(*t*, *f*, *A*, *σ*)|,

where *A *= 1.0, *σ*^2 ^= 10^-5^, *t *∈ [*τ*, *τ *+ *T*], *f *∈ [*f*_1_, *f*_2_].

The description of PWVD implementation is given in [42] and is implemented in a commercial software package BSIGNAL [56], both being developed by the present authors. Computational flowchart is given in the Appendix A [see Figure 15]. The WVD distribution satisfies the frequency marginal condition [52,54])

|X(ω)|2=12π∫−∞+∞WVDxx(t,ω)dt,
MathType@MTEF@5@5@+=feaafiart1ev1aaatCvAUfKttLearuWrP9MDH5MBPbIqV92AaeXatLxBI9gBaebbnrfifHhDYfgasaacH8akY=wiFfYdH8Gipec8Eeeu0xXdbba9frFj0=OqFfea0dXdd9vqai=hGuQ8kuc9pgc9s8qqaq=dirpe0xb9q8qiLsFr0=vr0=vr0dc8meaabaqaciaacaGaaeqabaqabeGadaaakeaacqGG8baFcqWGybawcqGGOaakiiGacqWFjpWDcqGGPaqkcqGG8baFdaahaaWcbeqaaiabikdaYaaakiabg2da9maalaaabaGaeGymaedabaGaeGOmaiJae8hWdahaamaapehabaGaem4vaCLaemOvayLaemiraq0aaSbaaSqaaiabdIha4jabdIha4bqabaGccqGGOaakcqWG0baDcqGGSaalcqWFjpWDcqGGPaqkcqWGKbazcqWG0baDaSqaaiabgkHiTiabg6HiLcqaaiabgUcaRiabg6HiLcqdcqGHRiI8aGqaaOGae4hlaWcaaa@517F@

where |*X *(*ω*)|^2 ^is the energy density spectrum, and *ω *= 2*πf *is the angular frequency. This equation means that the integral of the WVD over the time variable at a certain frequency *ω *yields the energy density spectrum of *x*(*t*) at this frequency. This property of the WVD is expanded here to compute the energy density spectrum for the HES format (part E1 of the format, outlined in Sect. 2.4).

D=|X(f)|2=∫ττ+T|PWVDxx(t,f)|dt
MathType@MTEF@5@5@+=feaafiart1ev1aaatCvAUfKttLearuWrP9MDH5MBPbIqV92AaeXatLxBI9gBaebbnrfifHhDYfgasaacH8akY=wiFfYdH8Gipec8Eeeu0xXdbba9frFj0=OqFfea0dXdd9vqai=hGuQ8kuc9pgc9s8qqaq=dirpe0xb9q8qiLsFr0=vr0=vr0dc8meaabaqaciaacaGaaeqabaqabeGadaaakeaacqWGebarcqGH9aqpcqGG8baFcqWGybawcqGGOaakcqWGMbGzcqGGPaqkcqGG8baFdaahaaWcbeqaaiabikdaYaaakiabg2da9maapehabaGaeiiFaWNaemiuaaLaem4vaCLaemOvayLaemiraq0aaSbaaSqaaiabdIha4jabdIha4bqabaGccqGGOaakcqWG0baDcqGGSaalcqWGMbGzcqGGPaqkcqGG8baFcqWGKbazcqWG0baDaSqaaGGaciab=r8a0bqaaiab=r8a0jabgUcaRiabdsfaubqdcqGHRiI8aaaa@5322@

The WVD also satisfies the time marginal condition [5]

|x(t)|2=12π∫−∞+∞WVDxx(t,ω)dω.
MathType@MTEF@5@5@+=feaafiart1ev1aaatCvAUfKttLearuWrP9MDH5MBPbIqV92AaeXatLxBI9gBaebbnrfifHhDYfgasaacH8akY=wiFfYdH8Gipec8Eeeu0xXdbba9frFj0=OqFfea0dXdd9vqai=hGuQ8kuc9pgc9s8qqaq=dirpe0xb9q8qiLsFr0=vr0=vr0dc8meaabaqaciaacaGaaeqabaqabeGadaaakeaacqGG8baFcqWG4baEcqGGOaakcqWG0baDcqGGPaqkcqGG8baFdaahaaWcbeqaaiabikdaYaaakiabg2da9maalaaabaGaeGymaedabaGaeGOmaidcciGae8hWdahaamaapehabaGaem4vaCLaemOvayLaemiraq0aaSbaaSqaaiabdIha4jabdIha4bqabaGccqGGOaakcqWG0baDcqGGSaalcqWFjpWDcqGGPaqkcqWGKbazcqWFjpWDaSqaaiabgkHiTiabg6HiLcqaaiabgUcaRiabg6HiLcqdcqGHRiI8aOGaeiOla4caaa@51BE@

Accordingly the integral of the WVD over the frequency variable at a certain time *t *yields the instantaneous signal power at that time. Using the energy density interpretation of the PWVD, the signal energy at time *t *and frequency *f *contained in a cell *dt *by *df *can be found as |*PWVD*_*xx*_(*t*, *f*)|*dtdf *[42]. Other important signal characteristics that can be defined from the PWVD include the instantaneous energy of the signal, or signal power [42]

*P*(*t*) = ∫|*PWVD*_*xx*_(*t*, *f*)|*df*.

Thus, the instantaneous energy of the heart sound signal, or the heart sound signal power, is computed for the HES format (part C1 of the format, outlined in Sect. 2.4) as

P(t)=∫f1f2|PWVDxx(t,f)|df, t∈[τ,τ+T]
MathType@MTEF@5@5@+=feaafiart1ev1aaatCvAUfKttLearuWrP9MDH5MBPbIqV92AaeXatLxBI9gBaebbnrfifHhDYfgasaacH8akY=wiFfYdH8Gipec8Eeeu0xXdbba9frFj0=OqFfea0dXdd9vqai=hGuQ8kuc9pgc9s8qqaq=dirpe0xb9q8qiLsFr0=vr0=vr0dc8meaabaqaciaacaGaaeqabaqabeGadaaakeaacqWGqbaucqGGOaakcqWG0baDcqGGPaqkcqGH9aqpdaWdXbqaaiabcYha8jabdcfaqjabdEfaxjabdAfawjabdseaenaaBaaaleaacqWG4baEcqWG4baEaeqaaOGaeiikaGIaemiDaqNaeiilaWIaemOzayMaeiykaKIaeiiFaWNaemizaqMaemOzaygaleaacqWGMbGzdaWgaaadbaGaeGymaedabeaaaSqaaiabdAgaMnaaBaaameaacqaIYaGmaeqaaaqdcqGHRiI8aOGaeiilaWIaeeiiaaIaemiDaqNaeyicI4Saei4waSfcciGae8hXdqNaeiilaWIae8hXdqNaey4kaSIaemivaqLaeiyxa0faaa@59C9@

The equation for Short Term Fourier Transform STFT is given by

STFTx(t,f)=∫−∞+∞x(t)h(t−τ)e−j2πftdt
MathType@MTEF@5@5@+=feaafiart1ev1aaatCvAUfKttLearuWrP9MDH5MBPbIqV92AaeXatLxBI9gBaebbnrfifHhDYfgasaacH8akY=wiFfYdH8Gipec8Eeeu0xXdbba9frFj0=OqFfea0dXdd9vqai=hGuQ8kuc9pgc9s8qqaq=dirpe0xb9q8qiLsFr0=vr0=vr0dc8meaabaqaciaacaGaaeqabaqabeGadaaakeaacqWGtbWucqWGubavcqWGgbGrcqWGubavdaWgaaWcbaGaemiEaGhabeaakiabcIcaOiabdsha0jabcYcaSiabdAgaMjabcMcaPiabg2da9maapehabaGaemiEaGNaeiikaGIaemiDaqNaeiykaKIaemiAaGMaeiikaGIaemiDaqNaeyOeI0ccciGae8hXdqNaeiykaKIaemyzau2aaWbaaSqabeaacqGHsislcqWGQbGAcqaIYaGmcqWFapaCcqWGMbGzcqWG0baDaaGccqWGKbazcqWG0baDaSqaaiabgkHiTiabg6HiLcqaaiabgUcaRiabg6HiLcqdcqGHRiI8aaaa@5850@

where *h*(*t*) is the analysis window function. The transform given by the Equation (18) with the Gaussian window function is called Gabor Transform. Since the STFT is complex-valued in general, the spectrogram is used for display purposes. The spectrogram is computed as the squared magnitude of the STFT:

Sx(t,f)=|STFTx(t,f)|2=|∫−∞+∞x(t)h(t−τ)e−j2πftdt|2
MathType@MTEF@5@5@+=feaafiart1ev1aaatCvAUfKttLearuWrP9MDH5MBPbIqV92AaeXatLxBI9gBaebbnrfifHhDYfgasaacH8akY=wiFfYdH8Gipec8Eeeu0xXdbba9frFj0=OqFfea0dXdd9vqai=hGuQ8kuc9pgc9s8qqaq=dirpe0xb9q8qiLsFr0=vr0=vr0dc8meaabaqaciaacaGaaeqabaqabeGadaaakeaacqWGtbWudaWgaaWcbaGaemiEaGhabeaakiabcIcaOiabdsha0jabcYcaSiabdAgaMjabcMcaPiabg2da9iabcYha8jabdofatjabdsfaujabdAeagjabdsfaunaaBaaaleaacqWG4baEaeqaaOGaeiikaGIaemiDaqNaeiilaWIaemOzayMaeiykaKIaeiiFaW3aaWbaaSqabeaacqaIYaGmaaGccqGH9aqpcqGG8baFdaWdXbqaaiabdIha4jabcIcaOiabdsha0jabcMcaPiabdIgaOjabcIcaOiabdsha0jabgkHiTGGaciab=r8a0jabcMcaPiabdwgaLnaaCaaaleqabaGaeyOeI0IaemOAaOMaeGOmaiJae8hWdaNaemOzayMaemiDaqhaaOGaemizaqMaemiDaqhaleaacqGHsislcqGHEisPaeaacqGHRaWkcqGHEisPa0Gaey4kIipakiabcYha8naaCaaaleqabaGaeGOmaidaaaaa@69DE@

### 2.4 Heart energy signature (HES) format

The HES format utilizes several additional averaged and instantaneous characteristics which may be extracted from the HES image and source signal. The components of the format to present heart sound energy are schematically illustrated in Figs. 1(A,B) and in the graphic form of Figs. 2(A,B). Figs. 1(A,B) illustrate schematically generic PCG (oscillographic heart sound display) of two heart beats and its HES spectrogram reflection. It includes S1, S2, S3 sounds and also illustrate S2 split as well as systolic and diastolic intervals. Fig. 1B demonstrates (maps) heart sound components as energy contours, with shape depending upon energy distribution in time and frequency. Thus, additional heart sounds or murmurs as well as normal S1 and S2 will be manifested as additional energy contours and missing sounds will lead to significant reduction or complete disappearance of these contours. Contour values inside the dark zones always exceed certain predefined energy threshold, for example 20% of maximum energy or 50% of maximum energy.

**Figure 3:**
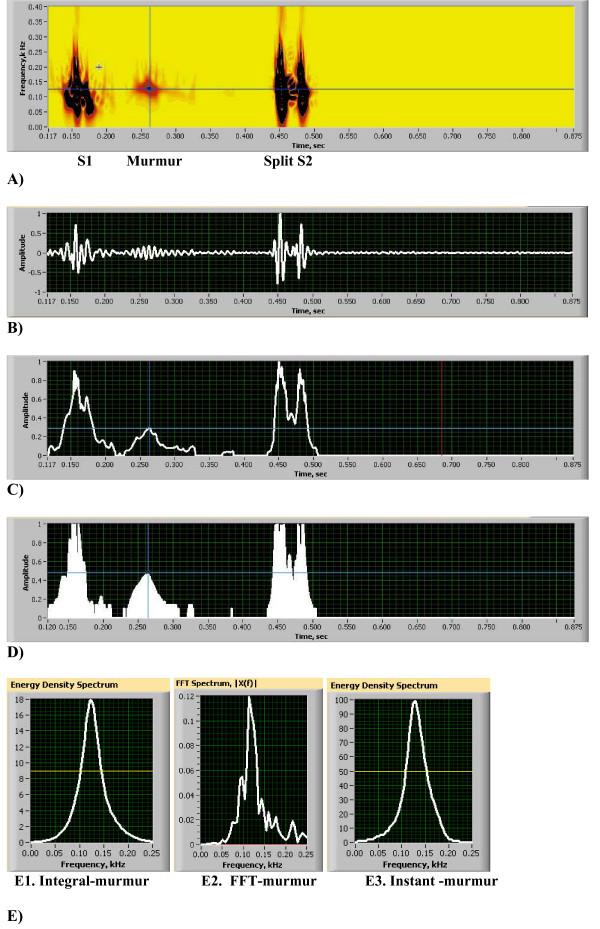
(A-E). Heart Energy Signature (HES) Format and Display [see Additional file 2]. A) Heart Energy Signature Spectrogram, cross hair shows locations of instantaneous extraction lines (A1: Eq. 13). B) Signal Plot (B1: Eq. 14). C) Normalized square root of Power (C1: Eq.15a). D) Instantaneous square root of Power, frequency = 107 Hz (D1: Eq.15b). E) Frequency Distributions (E1: Eq. 16). E2. Peak frequency 124 Hz, High frequency 133 Hz, Low frequency 111 Hz, half-bandwidth 11 Hz. E3. Peak frequency 128 Hz, High frequency 153 Hz, Low frequency 107 Hz, half bandwidth 23 Hz. All measured at 50% of maximum amplitude.

**Figure 4:**
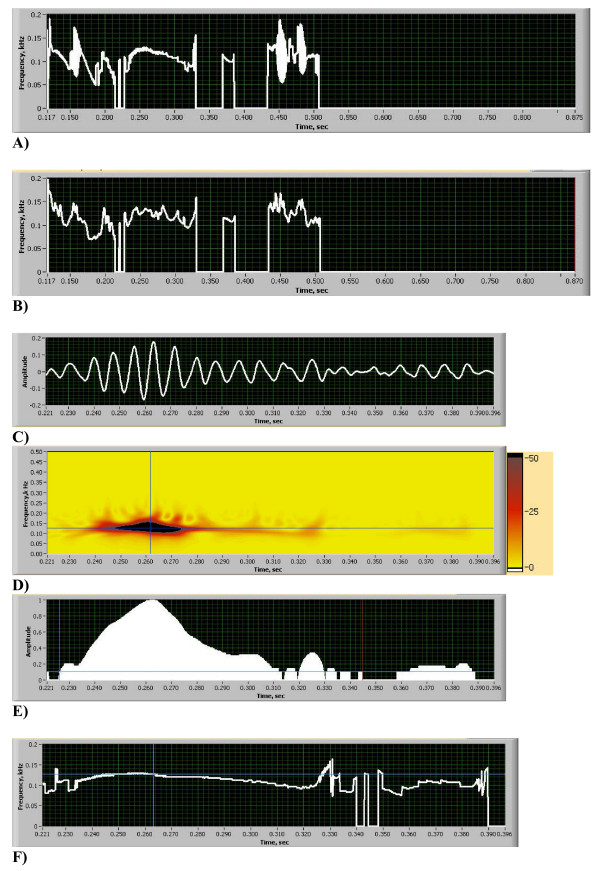
(A-F). Heart Energy Signature (HES) Format and Innocent Murmur Details [see Additional file 2]. A) Instantaneous Peak Frequency (IPF) extracted from HES (F1: Eq. 17). B) Instantaneous Mean Frequency (IMF) extracted from HES (F1: Eq. 18). C) Murmur Signal Plot. D) Murmur HES. E) Murmur normalized square root of power, time duration is measured 118 ms, using 10% threshold value. F) Murmur IPF Plot. At time t = 0.263 s frequency is measured equal to 127.1 Hz.

In the majority of heart conditions a single heart beat is sufficient to define format. Let us assume that the heart beat is recorded during the time interval [*τ*, *τ *+ *T*] with measurement instrument capable of capturing frequency range [*f*_1_, *f*_2_]. Thus, HES format includes (see also Figs. 3(A–E) and Figs. 4(A–B)):

**Figure 5:**
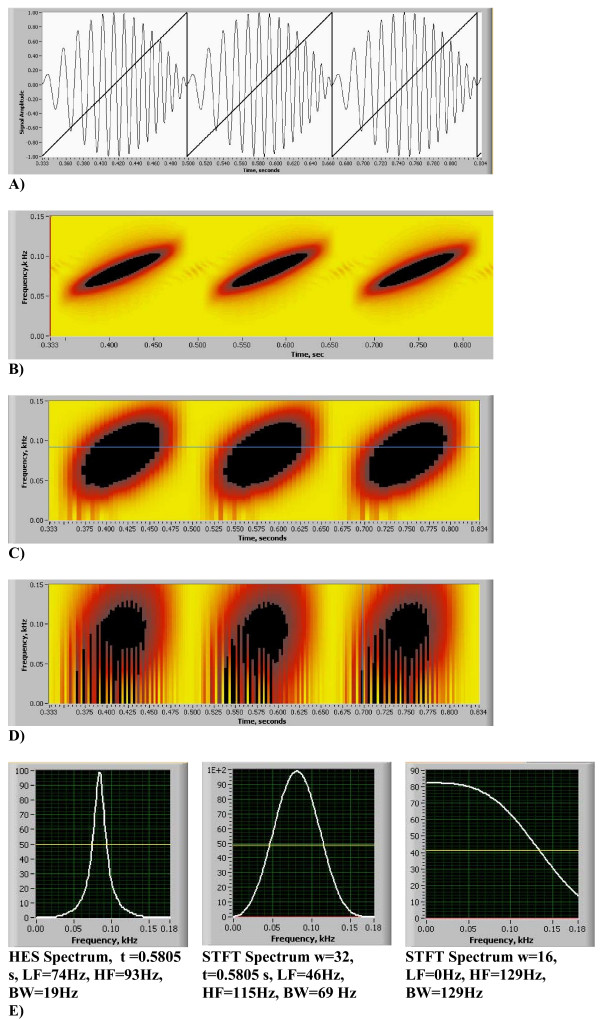
(A,B,C,D,E). Comparison of HES and STFT frequency resolution on the spectrogram, chirp function. A) Three pulses of normalized chirp function, linear change in frequency is shown by the darker line, on this line "-1" frequency corresponds with 60 Hz and "+1" corresponds with 109.8 Hz. B) HES Energy Signature Spectrogram obtained using present method. C) Spectrogram obtained using STFT with window size w = 32. D) Spectrogram obtained using STFT with window size w = 16. Same color scale was utilized all three figures. E) Comparison of Frequency Resolution in the energy density spectrum between HES and STFT given for the same signal and moment of time (LF - low frequency, HF = high frequency, BW - bandwidth, all measured at 50% of maximum amplitude).

**Figure 6:**
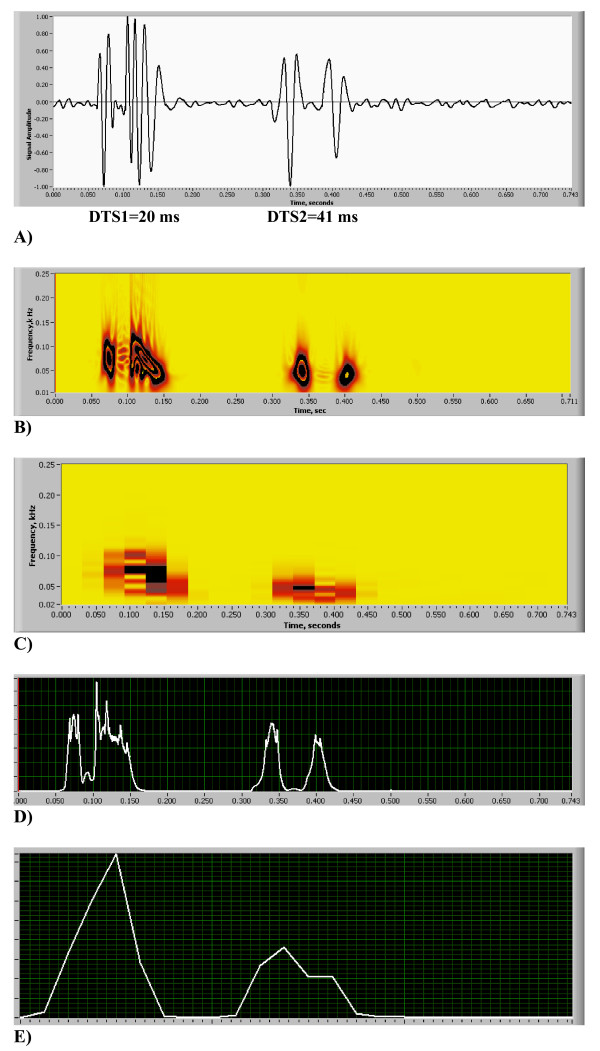
(A,B,C,D,E). Comparison of HES and STFT frequency resolution on the spectrogram, simulated S2 split function [see Additional file 3]. A) Phonocardiogram: two realistic S2 narrow split test. B) HES Spectrogram (two separate energy contours are clearly seen). C) STFT w = 256 Spectrogram (separate counters united together, split is lost). D) HES Integral Power Plot showing excellent split resolution accuracy at mid point DTS1 = 14.6 % of the peak, DTS2 = 2.9% of the peak. E) STFT Spectrogram w = 256 Power Plot (split is completely lost) accuracy at mid point DTS1 = 78.5% of the peak, DTS2 = 66% of the peak.

**Figure 7:**
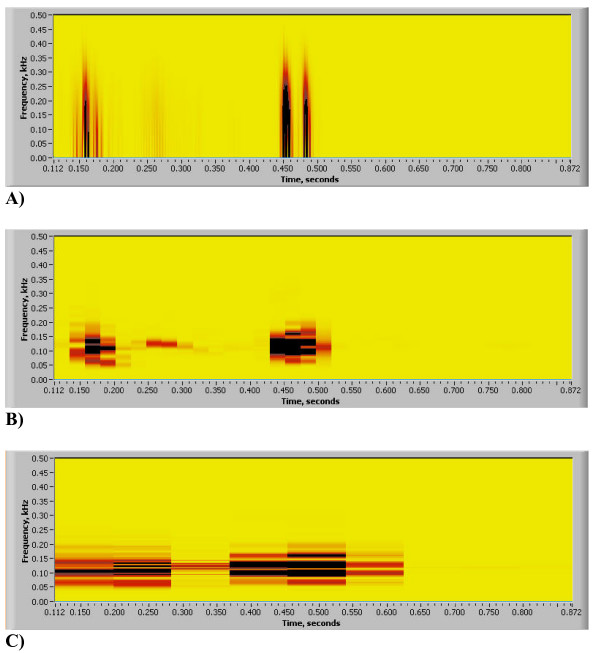
(A,B,C). STFT Heart Sound Resolution (same heart sound that is shown in Figs. 3(A-E)). A) STFT, time window w = 16, frequencies are overstretched, time resolution is good. B) STFT, time window w = 256, frequencies are crude, time resolution is compromised (no split), image is pixilated. C) STFT, time window w = 1024, frequency resolution is good, time resolution is completely compromised, image is pixilated.

**Figure 8:**
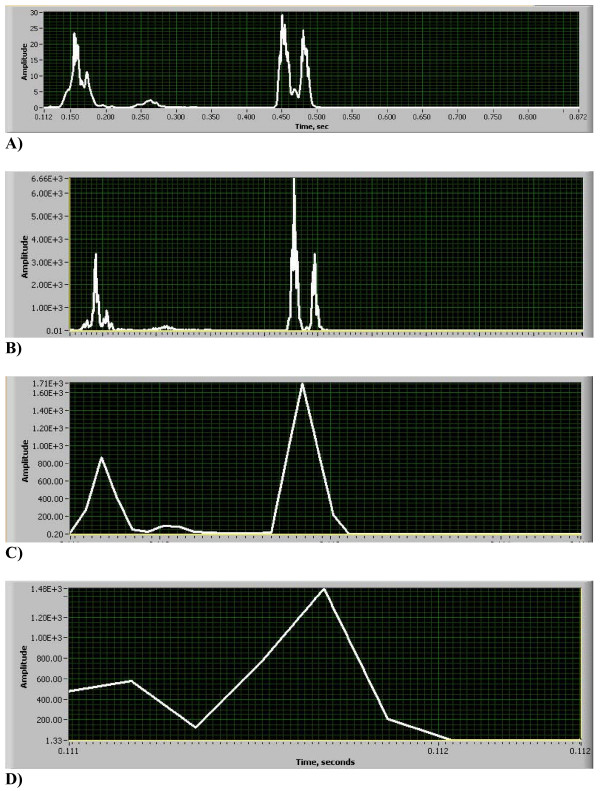
(A, B, C, D). STFT Heart Sound Time Resolution (same heart sound that is shown in Figs. 3A and B, sampling rate 11 kHz). A) Power Plot HES. B) Power Plot STFT, window w = 16. C) Power Plot STFT, window w = 256. D) Power Plot STFT, window w = 1024.

**Figure 9:**
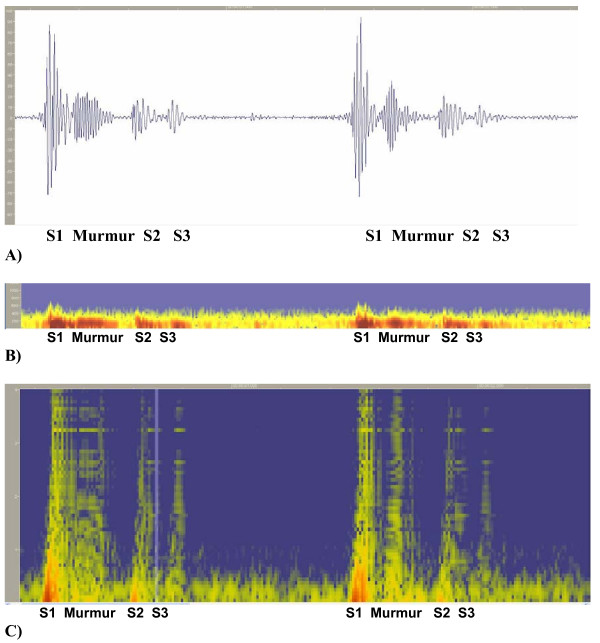
(A,B,C). Spectrogram Method signal resolution. A) Original Recording (PCG). Displayed using Meditron Analyzer software (Welch-Allyn, NY). [See Additional file 1]. B) Spectrogram obtained using Nero Wave Editor Software. C) Wavelet Scaleogram (obtained using wavelet transform, Nero Wave Editor Software).

**Figure 10:**
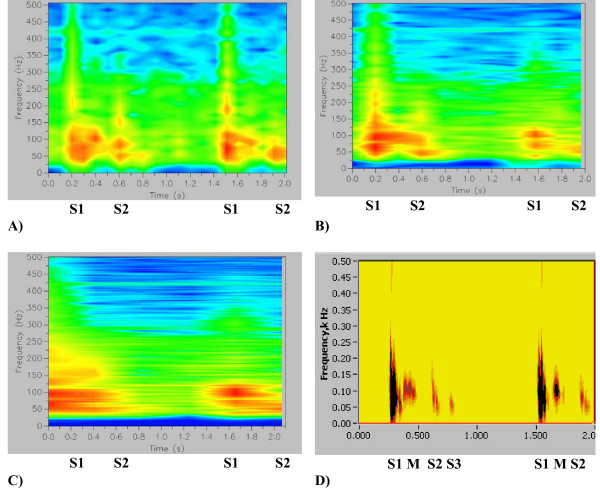
(A, B, C, D). Changes in the STFT Spectrogram due to change in the window size. PCG is shown on Fig. 9A, Spectrograms shown on A), B) and C) were obtained using Meditron Analyzer software distributed by Welch-Allyn, NY. Also compare against the Fig. 2 obtained using HES method. [see Additional file 1]. A) Window size w = 1024 points (better time resolution). B) Window Size w = 2048 points. C) Window Size w = 4096 points (better frequency resolution). D) HES spectrogram (see also in greater detail in Figs. 2(A-C)).

**A1. **The distribution of the heart sound energy simultaneously in time and frequency

*E *= *E*(*t*, *f*), *t *∈ [*τ*, *τ *+ *T*], *f *∈ [*f*_1_, *f*_2_]

where *E *is heart sound energy distribution, *t is *time, *f *is frequency.

**B1. **The normalized heart sound corresponding to the heart sound energy

*x *= *x*(*t*), *t *∈ [*τ*, *τ *+ *T*], *x*(*t*) ∈ [*-*1, +1]

**C1. **The instantaneous energy of the heart sound signal, or heart sound power, corresponding to *x*(*t*)

*P *= *P*(*t*) *t *∈ [*τ*, *τ *+ *T*]

**D1. **Instantaneous heart sound power, corresponding to *x(t) *at a particular frequency *f*

*P *= *P*(*t*) *t *∈ [*τ*, *τ *+ *T*]

**E1. **The energy density spectrum of the heart sound, corresponding to *x*(*t*)

*D *= |*X*(*f*)|^2^, *f *∈ [*f*_1_, *f*_2_]

**F1. **Instantaneous mean and peak frequency of the heart sound signal, corresponding to *E(t,f)*. Instant peak frequency (IPF) = frequency f* for given t*, for which

*E*(t*,f*) = Max(*E*(t*,*f*))

Instant mean frequency, or IMF, defined as

f*=∫f0f1E(t*,f)*fdf∫f0f1E(t*,f)df
MathType@MTEF@5@5@+=feaafiart1ev1aaatCvAUfKttLearuWrP9MDH5MBPbIqV92AaeXatLxBI9gBaebbnrfifHhDYfgasaacH8akY=wiFfYdH8Gipec8Eeeu0xXdbba9frFj0=OqFfea0dXdd9vqai=hGuQ8kuc9pgc9s8qqaq=dirpe0xb9q8qiLsFr0=vr0=vr0dc8meaabaqaciaacaGaaeqabaqabeGadaaakeaacqWGMbGzdaahaaWcbeqaaiabcQcaQaaakiabg2da9maalaaabaWaa8qCaeaacqWGfbqrcqGGOaakcqWG0baDdaahaaWcbeqaaiabcQcaQaaakiabcYcaSiabdAgaMjabcMcaPiabcQcaQiabdAgaMjabdsgaKjabdAgaMbWcbaGaemOzayMaeGimaadabaGaemOzayMaeGymaedaniabgUIiYdaakeaadaWdXbqaaiabdweafjabcIcaOiabdsha0naaCaaaleqabaGaeiOkaOcaaOGaeiilaWIaemOzayMaeiykaKIaemizaqMaemOzaygaleaacqWGMbGzcqaIWaamaeaacqWGMbGzcqaIXaqma0Gaey4kIipaaaaaaa@548F@

The HES can be stored as a digital file and displayed visually. Its visual representation consists of a following set of quantitative plots and images (Figs. 3(A–E), Figs. 4(A,B)):

• a two-dimensional image (2D) representing the distribution of the heart sound energy simultaneously in time and frequency as defined in A1 (Fig. 3A)

• a time plot of the normalized heart sound corresponding to the heart sound energy as defined in B1 (Fig. 3B)

• a plot of the instantaneous energy of the heart sound, or heart sound power, as defined in C1 (Fig. 3C)

• a plot of the instantaneous energy of the heart sound at a given frequency, or heart sound power at a given frequency, as defined in D1 (Fig. 3D)

• a plot of the energy density spectrum of the heart sound, as defined in E1 (Fig. 3E).

• a plot of the time averaged energy density spectrum computed by Fast Fourier Transform FFT (Fig. 3E2)

• a plot of the instantaneous peak (Fig. 4A) and mean (Fig. 4B) frequencies, as defined in F1

These plots help to provide a comprehensive description and quantitative differentiation of heart abnormalities. For example, Fig. 3A provides qualitative visualization of every cardiac sound event, including S2 split and gives instantly visual ranges of change in frequency and time. Fig. 3C provides us with precise estimation of S2 split duration (time distance between peaks is equal to 30 ms) and S2 duration (75 ms) and provides relative scale of intensity for systolic murmur (30% of maximum power). Fig. 3D provides the same measure, but at the dominant murmur frequency of 125 Hz (horizontal line on Fig. 3A). On that frequency murmur power is increased to 45% of maximum. Fig. 3E1 shows the energy density spectrum of murmur obtained from HES for entire murmur time duration (murmur peak is at 125 Hz and half-bandwidth at 50% magnitude is 21 Hz), same spectrum obtained by FFT is shown on Fig. 3E2 (peak frequency 124 Hz, half-bandwidth 11 Hz and on Fig. 3E3 we show instant energy density spectrum obtained at murmur intensity peak (vertical line on Fig. 3A), showing peak at 128 Hz and half-bandwidth of 23 Hz. This instantaneous frequency distribution can be obtained at any time instant for murmur or any other sound component. We also measure effective frequency bandwidth (lower frequency LF and high frequency HF) at 50% of the magnitude on the spectrum distribution. Instantaneous peak and mean frequencies extracted from HES are shown on Figs. 4(A,B) where we can clearly see dominant frequencies of S1, S2 and murmur. Murmur frequency varies in time between 100 to 150 Hz; S2 frequency varies between 160 to 100 Hz and S1 changes between 70 to 145 Hz. Instantaneous jumps in frequency can be visualized, measured and correlated with extra sounds(clicks, snaps, splits, etc). To further illustrate HES format ability to characterize heart sound we extracted murmur signal from the heart sound (Fig. 4C) and analyzed it separately. Murmur energy signature is depicted in Fig. 4D showing narrow frequency width, with data extraction performed at vertical and horizontal lines. From this image mean frequency of 125 Hz, frequency half-bandwidth 20 Hz, lower frequency 102 Hz and high frequency 142 Hz are measured. On Fig. 4E the murmur instantaneous power change at dominant frequency is shown. The horizontal line drawn at 10% power threshold shows murmur start, end points and timing of murmur energy peak (0.262 sec). Fig. 4F shows instantaneous murmur frequency variation, with frequency being 125 Hz at murmur energy peak.

### 2.5 Heart sound data

In developing this method heart sounds that were recorded between 1980–91 by one of the authors at the Department of Pediatric Cardiology, IWK Center at Dalhousie University, Canada were utilized. Heart sounds were collected at four major auscultation positions, and subsequently documented as a database which included auscultation position, binary wave sound track (recorded at 11 kHz sampling rate), clinical diagnosis and auscultatory diagnosis. Most of this data was subsequently included in the educational teaching system, EarsOn [50] which included 260 clinical recordings and which was successfully utilized in the teaching of medical students and physicians [3]. All heart sound recordings used in this paper are from that database (see Additional files 1, 2, 5, 6, 7, 8, 9), and no artificial or simulated sounds have been used.

## 3.0 Results and Discussion

### 3.1 Overview – what can we do with HES

New hardware and digital sound recording technologies can not completely revitalize old approaches (i.e. PCG or spectrograms) unless new methods present new advantages. The HES spectrogram offers a variety of new tools that may greatly reduce all existing disadvantages of PCG (see Sect. 1.2). By allowing dynamic separation of signal contributions (tones) in frequency and space intrinsic details of signal morphology are demonstrated. The HES spectrogram does not require special filtration. Background and line noise equally contribute to the entire spectrogram and thus are effectively eliminated from the visual image. The time dependent heart power plot deciphered from the HES spectrogram allows easy identification of start and end points of various heart sound components, showing as well separate peak components when signals are split. The method allows objective characterization of both time-averaged and instantaneous murmur's mean and peak frequencies and effective bandwidth, thus aiding in subsequent murmur classification. Folded multi-component frequencies are immediately visualized and documented on the HES image (Eq. 13). Integral HES energy (power plot, Eq. 15a) allows provision of a very detailed characterization of murmur and heart sound intensity (as it changes with time), offering unlimited possibilities to introduce precise digital scores to replace subjective Freeman-Levine system [59] of murmur intensity grades. Finally, the combination of PCG, HES image, power and frequency plots (Eqs. 13–18), allow much easier separation and identification of heart sounds, without the necessity of additional synchronized ECG tracings. These data plots also offer precise ability to measure timing of cardiac sound events, durations, delays and documentation of arrhythmias (variation in distances between energy peaks) if present. Additionally, HES offers the possibility of correlating time varying frequency (Eqs. 17–18) and intensity (Eqs. 15 (a,b)) of heart sounds with time dependent C-Doppler velocity profiles or M-mode ultrasound. This is done by a virtue of providing precisely calculated time varying mean frequency, instantaneous frequency and signal energy derived from the HES image. Rabin [63] directly correlates murmur intensity and murmur frequency with velocity and pressure gradient through an obstruction.

### 3.2 Examples of innocent murmurs using HES spectrograms

An example of a HES spectrogram for the innocent Still's murmur is presented in Fig. 2A with additional details shown in Figs. 2(B,C). The key characteristic of this image is the sharp resolution, making it possible to visually identify every component of the heart sound in a manner schematically described in Figs. 1(A,B). This heart sound is recorded at the apex. S1 is followed shortly by a 2+/6 systolic murmur of distinct musical quality. S1, S2 and S3 sounds are easily heard, and S2 sound is single to the experienced auscultator. However HES indicates narrow splitting of A2 and P2, illustrating the capability of the method. To illustrate the quantitative side of this method, instantaneous mean frequency characteristics were measured for murmur at times t = 0.4 s; 0.43 s; 0.45 s and 0.47 s. Murmur mean frequency and half-bandwidth were correspondingly 112.9/13.4; 106.2/14.8; 99.5/10.7; 94/13.4 (all measured in Hz) all consistently demonstrating narrow frequency bandwidth.

A second example of an innocent murmur recording of a pediatric patient is shown in Figs. 3(A–E) and 4(A–F). Sound recorded at 2^nd^ LSB. These figures contain 13 components and demonstrates full graphic representation of HES image and format. The first five key elements are shown one below the other. The HES image shows corresponding flooded energy contours (Fig. 3A). The PCG of a single heart beat shows S1, murmur and split S2 sounds in a consecutive order (Fig. 3B). Power plots of Figs. 3C and 3D illustrate the most significant energy components and their gradients. Fig. 3C demonstrates the integral power plot (energy integrated across all frequencies) and in Fig. 3D power is extracted at a pre-selected murmur frequency (shown as a horizontal line at the cross-hair, Fig. 3D), The power plot clearly shows the maximum ratio of murmur to S2 intensity to be equal to 32%, which is consistent with clinical impression of the murmur being of 2+/6 intensity [50]. The instantaneous frequency plot (Figs. 4A and 4B) illustrates the start and end of each heart sound. The variation of frequency of these components is also seen. Clearly demonstrated is that the murmur frequency decreases, that S3 frequency is the lowest; and that S2 has two frequency components separated in time.

The following three components are presented in Fig. 3E and represent:

1) energy density spectrum of the heart murmur (E1),

2) murmur time averaged frequency spectrum, obtained using FFT (E2), and

3) instantaneous energy density spectrum of heart murmur at its peak intensity (vertical cross-hair at the Fig. 3A).

Statistical characteristics of murmur frequency spectrum (peak frequency, mean value, bandwidth around mean value) and all numerical frequency criteria are obtained using equations (17–18) by integrating the energy signature image. Corresponding murmur detail and its HES are shown in Figs. 4C and 4D. Murmur oscillations are evident (basically non-musical), the frequency band of which is shown in detail, confirming the clinical diagnosis of innocent flow murmur [39,50].

In Figs. 4D and 4E, "zoomed in" details of the HES are demonstrated, as well as detail of Doppler echo-like murmur intensity variation plot. The detail of the instantaneous murmur frequency plot (Fig. 4F), showing the murmur gradually increasing its frequency from 80 Hz to 125 Hz and then reducing it to 70 Hz, is also shown. Easily seen is that while time averaged frequency and instantaneous time frequency bands are narrow, the time dependent variation of the frequency spectrum of the murmur is significant. Abrupt start and end points of the murmur are clearly evident in these plots.

### 3.3 STFT spectrogram accuracy vs. new method

Classic spectrograms display frequency on the vertical axis and time on the horizontal, and plot sound intensity (measured in decibel) as a color map. They utilize Short Term Window Fourier Transform (STFT), which is a first order method and is subject to the uncertainty principle, whereby one cannot achieve simultaneous resolution in both frequency and time [32]. Publications concerning signal processing also point to the inadequacy of the STFT method [28-30].

#### 3.3.1 Comparison using model signals

In Figs. 5(A–E) comparison of the accuracy of newly developed HES method vs. STFT spectrogram method (Eqs. 12a and 12b) is shown. Labview V7.0 (National Instruments, Austin, TX) was utilized for STFT analysis with Hanning time window (w). In all cases frequency resolution window was set to be 2048 points. On Fig. 5A we present our test signal – basic chirp (6 kHz sampling rate) that emulates linear change in frequency between 60 Hz and 110 Hz (Eq.19). Similar test results are also presented in [58]. Shown also are: HES energy signature spectrogram in Fig. 5B; two examples of STFT spectrograms with time windows w = 32 and 16 data points in Figs. 5(C,D) and quantitative frequency resolution comparison in Fig. 5E. We conclude that HES frequency resolution at signal peak is +-9.5 Hz and is 3.7 to 6.78 times better than STFT resolution. At signal peak HES mean frequency estimate 82.11 Hz matches very well (3%) with analytical chirp frequency of 84.9 Hz. Accuracy of temporal resolution was studied using model function derived from real heart sounds (Fig. 6A, sampling frequency 11 kHz). This function [see Additional file 3] includes two events (DTS1 and DTS2), each event presenting a narrow time split cardiac sound. DTS1 has 20 ms split and DTS2 has 41 ms split. HES results are presented on Fig. 6B and STFT (w = 256) results are presented on Fig. 6C. Matching power plots are presented on Figs. 6D(HES) and 6E (STFT). Clearly seen is that HES provides excellent visual separation of both temporal events, and time split is evident and measurable on energy contour plots and integral power plot. STFT spectrogram images are smeared, there is no visual separation and split is lost on the power plot. HES temporal accuracy at split mid point is 14.6% for DTS1 and 2.9% for DTS2, while correspondingly STFT temporal error is 78.5% and 66%.

In Figs. 7(A,B,C) STFT spectrograms that correspond to the HES image of Fig. 3A are shown. Clearly seen is that short window (Fig. 7A) overstretches frequency range, medium size window (Fig. 7B) provides very crude visual resolution and large size time window (Fig. 7C) completely smears all important components of the heart sound. Matching integral power plots obtained using Eq. 15a are presented in Figs. 8(A,B,C,D). Shown also is the excellent HES resolution of S2 time split (Fig. 8A), excellent time resolution of short window STFT (Fig. 8B), the bad time resolution for medium size STFT window (S2 split is lost, Fig. 8C) and the poor time resolution for large size STFT window (S1,S2 and murmur are smeared together, Fig. 8D). Corresponding quantitative frequency resolution measures are presented in Table 1. Again seen is that frequency resolution for STFT time windows w = 256 & 1024 is good, but image quality is pixilated, and time resolution for these windows is grossly insufficient (Figs. 8C and 8D). Time resolution for STFT window w = 16 is excellent, but frequency resolution is grossly overestimated, lower bound of frequency is completely lost (zero Hz), upper bound is exaggerated by 41% and frequency bandwidth is exaggerated by 215%.

#### 3.3.2. Comparison using clinical heart sound and third party software

In this section comparison of the STFT spectrogram method with our newly developed method using example of a clinical heart sound is carried out. The original recording is also presented in Figs. 3(A–E) and 4(A–F) (using new method), the heart sound recording being that of an innocent murmur (musical) of a child, the sound track being recorded with 11 kHz sampling rate. To enable further research and comparisons the sound track is attached [see Additional file 2]. The STFT spectrograms shown were obtained using state-of-the art Meditron Analyzer software (FDA cleared clinical product distributed by Welch Allyn, NY) using its default (best) transformation option – Hanning window with 1024, 2048, 4096 data points correspondingly. A typical clinical complaint concerning this method is that clear separation of heart sounds is not allowed, and that precise identification of A2 and P2 components and their split is difficult. In Figs. 9(A,B) and 10(A,B,C), spectrograms obtained using STFT are shown, which currently have been the only means of obtaining dynamic content of the heart sound spectral signal. Quantitative data extraction is not possible as it is not possible using current third party software.

Recent studies [16-19,23-25] show similar spectrograms, also without presenting quantitative details. Certain researchers (i.e. Bentley et al [55]) simply select window function and its parameters (i.e. length) for a particular heart signal by trial and error. This leads to ambiguity in time-frequency resolution to the point that two STFT computed for the same signal, but with different window function parameters, could hardly be identified as computed for the same signal This is clearly demonstrated by comparison of Fig. 7A and Fig. 7C). Results presented in Figs. 10(A,B,C) illustrate this ambiguity as it is not clear which resolution is correct. Window sizes w = 1024, 2048, 4096 data points with sampling rate of 11 kHz were employed. Small size window will improve time resolution, but will have poor frequency resolution and vise versa.

A recording of an innocent murmur of a child (Fig. 9A) is used to demonstrate the drawbacks of the wavelet method. The spectrogram detail is shown in Fig. 9B, while Fig. 9C illustrates the wavelet transform based scaleogram approach similar to one used by Kim and Tavel [24]. Wavelet results were obtained using commercial music editing software Nero Wave Editor and are presented here for illustration purposes. While some correspondence between waveforms and wavelet scaleogram "splashes" is seen, detailed resolution remains insufficient and the image becomes highly smeared, especially at lower frequency range. This behavior is typical for wavelet transform and similar appearing visual images have been reported [27,30]. It has been indicated [62] that STFT and wavelet transform have similar resolution and that improved localization of acoustic events will be useful. Wavelet based visual images (contours) were also reported by Tovar Corona et al. [20,21], however the article presents very sketchy results and method description. It is well established in the signal processing literature [28,30,54] that the STFT spectrogram image is window size dependent, as is clearly seen in the Figs. 10A,B, and 10C. As window size (number of computational points in the sample) increases, the time resolution of the spectrogram decreases (increased horizontal smearing), resulting in improved frequency resolution. These trends are mutually exclusive. Separating the murmur from S1 is very difficult, and detecting S3 with certainty is virtually impossible, as in Fig. 10A, which initially appeared satisfactory, Figs. 10B and 10C become unusable. Close analysis of spectrograms (Figs. 10A, 10B, 10C)) also indicates the tendency to overestimate the upper frequency bound of the signal, especially when improved time resolution is sought. This in turn leads to unrealistic and significantly overestimated conclusions about murmur frequencies. The HES method based spectrogram of the same recording is shown in Fig. 10D and in greater detail in Figs. 2A and 2B. HES is the second order method and simultaneously resolves both time and frequency and separate all key components of the heart sound. This can be seen from Fig. 2A. Fig. 2B clearly demonstrates the detail of Fig. 2A, focusing on the murmur at the end of S1, and on S2 and S3 sounds. Note a clear separation of the heart murmur, and that aortic and pulmonary components of P2 are clearly visible. One also sees in S1 clear separation of mitral and tricuspid components in the power (energy) vs. time plot (Fig. 2C). We can also identify from the plot time the delay between S2 and S3 as well as their duration. In Table 2 we provide further quantitative comparison of murmur, S1 and S2 time averaged frequency resolution using HES and STFT (obtained by the authors using method described in Sect 3.3.1 and Eqs. 12a and 12b, time window w = 16 data points, frequency window 2048 data points). STFT exceeds upper frequency estimation by 60 to 66% and frequency bandwidth estimation between 2.2 to 5 times.

Several other relevant issues and especially end point detection using HES, PCG, STFT and effects of filtration are discussed in the Additional file 4.

### 3.4 Analyzing heart energy signature

**Figure 11:**
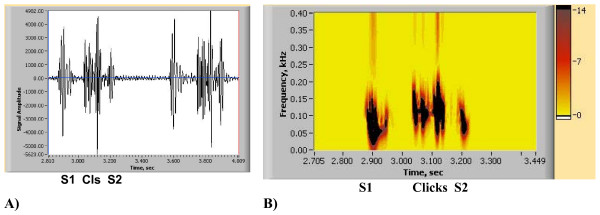
(A, B). Abnormal Heart Sound of Mitral Valve Prolapse (3 clinical clicks) [see Additional file 5]. A) PCG (2 heart beats). B) HES (single heart beat).

**Figure 12:**
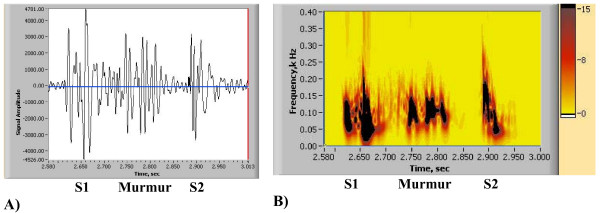
(A, B). Abnormal Heart Sound of Aortic Stenosis (single heart beat) [see Additional file 6]. A) PCG (single heart beat). B) HES (single heart beat).

**Figure 13:**
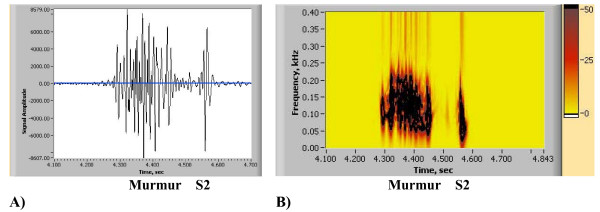
(A, B). Abnormal Heart Sound of Pulmonary Stenosis (single heart beat) [see Additional file 7]. A) PCG (single heart beat). B) HES (single heart beat).

**Figure 14:**
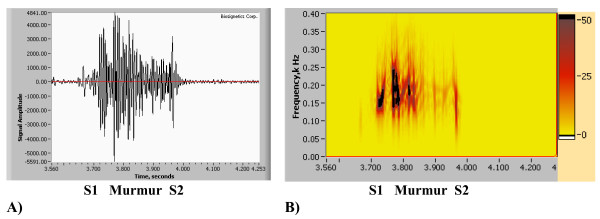
(A, B). Abnormal heart sound of Severe Tetralogy of Fallot (acyanotic) [see Additional file 8]. A) PCG (single heart beat). B) HES (single heart beat).

One common and distinctive feature for most heart abnormalities is the appearance of additional energy contours, as depicted in Fig. 1B. Here S1 and S2 contours are "normal" (responsible for typical lub-dup sound) and additional contours may indicate murmurs, and extra sounds. For most of these circumstances (additional dark contours) evaluation by the cardiologist will be required. Figs. 11(A,B), 12(A,B), 13(A,B) and 14(A,B) show PCGs and corresponding HES for cases of heart disorders demonstrating systolic clicks and murmurs. Figs. 11(A,B) illustrate mitral valve prolapse (MVP) with three clicks being elicited on cardiac auscultation. The HES figure shown in Fig. 7B clearly indicates 3 extra energy contours located between S1 and S2 in late systole and which are on the left and right hand side correspondingly. Detecting the exact number of clicks by auscultation is a challenging task. Figs. 12A and 12B present the case of aortic stenosis of moderate severity. Fig. 12B illustrates the appearance of a complex energy contour (consisting of 3 components) between S1 and S2, with S1 having two components, the second of which represents an aortic ejection click. Note that the S2 energy contour shows higher frequency at first (A2 component), followed by lower frequency P2 component. The murmur occurs in the middle of the systolic interval, with high frequency not exceeding 150 Hz. Note, that PCG (Fig. 12A) does not show clear separation of S1 from the murmur, while HES demonstrates a clear time border. In the murmur of pulmonary stenosis, as displayed in Figs. 13A, S1 is not seen. This becomes more evident in Fig. 13B. The murmur is represented by a long and wide energy contour, with high frequencies reaching 230 Hz, correlating with the high degree of obstruction that was clinically diagnosed for that patient [50]. The murmur begins early in systole, ending before S2. Strong correspondence between the pressure gradient (obstruction) and murmur frequency was also demonstrated clinically by other authors [60,61,63]. Figs. 14(A,B) demonstrate an example of Tetralogy of Fallot. This murmur is not associated with post-stenotic valvar dilatation. The murmur has a similar appearance with pulmonary stenosis (Figs. 13(A,B)), but is longer and occupies entire systole, with frequencies reaching 250 Hz. S1 sound is also clearly seen.

**Figure 15:**
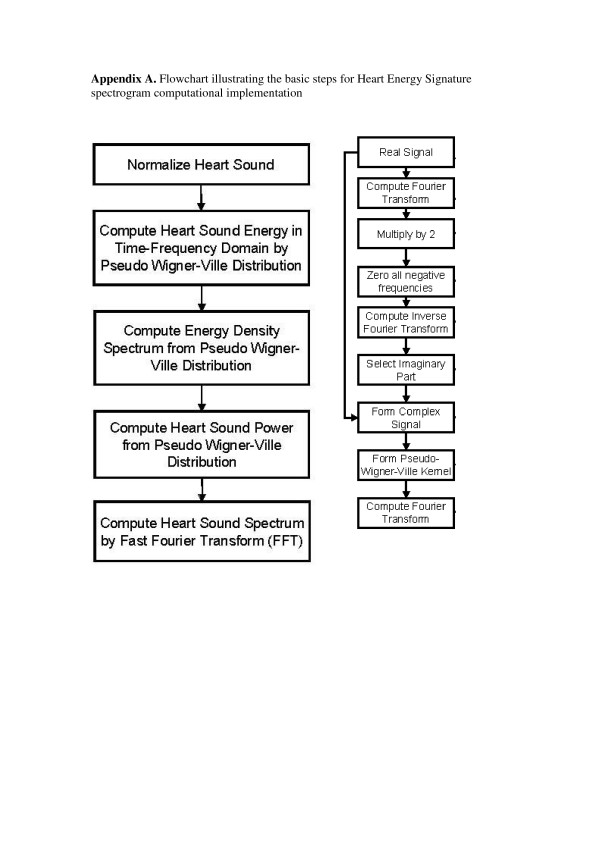
Flowchart illustrating the basic steps for Heart Energy Signature spectrogram computational implementation.

We conclude that abnormalities may be detected using overall and immediate visual contrast i.e. self-referencing property of HES image. By also utilizing the quantitative nature of the above displays, the ability to demonstrate murmur pitch, for example, is shown.

## 4.0 Future Work

Future work will focus on documenting various important characteristics of HES spectrograms and specifically on their ability to characterize heart murmur frequency, S2 heart sound frequency and its split, murmur timing and duration, murmur intensity, S3 sound presence and intensity, S1 presence and intensity, presence of ejection sounds and arrhythmias. The library of heart sounds of one of the authors [3,50] will be utilized to conduct these studies. The systolic ejection click is a very difficult auscultatory event for the physician to elicit, yet may be the single abnormal finding. One study currently in progress is to study a cohort of patients with a heart murmur to determine blindly whether the murmur is innocent or abnormal. A study of patients with systolic ejection clicks by HES is in process. The ultimate goal is to have available a low price bedside automated diagnostic tool to supplement the existing problem of low auscultatory skill of the family physician.

## Conclusion

In this paper a new method and format of analyzing heart sounds using Cohen class joint time-frequency transformation is presented. Initial results of applying these methods to normal and abnormal heart sounds are demonstrated. This method allows detailed quantitative characterization of heart sounds and uses both visual images and a system of integral plots that characterize time averaged and instantaneous sound intensity and frequency variations. Examples of the innocent murmur, mitral valve prolapse, pulmonary and aortic stenosis, and Tetralogy of Fallot are presented. All heart sound components, i.e. S1, S2, S3, murmurs and sound splits, were clearly separated in time and frequency. High resolution of generated heart sound images, in both time and pitch, are demonstrated, presenting a distinctly improved quality to traditional spectrogram images (based on SFFT). The resulting visual images have self-referencing quality, whereby individual features and their changes become immediately obvious.The procedure also offers a new parameter for cardiac research, which, for example by virtue of its ability to portray third heart sounds [58], may well play a valuable part in the management of patients with coronary artery disease. Current ACC/AHA Guidelines recommend that patients with unstable angina and a concurrent auscultated S3 be classified in the group at highest risk for adverse outcomes and considered candidates for an early invasive strategy [58].

## Supplementary Material

Additional file 1 Innocent Still's Murmur 1. Binary soundtrack of an innocent murmur used in Figures 2 and 10. Heart sound is recorded at the apex. Click here for file

Additional file 2 Innocent Stills Murmur 2. Binary soundtrack of an innocent murmur used in Figures 3 and 4. Heart sound is recorded at LSB, 2nd space. Click here for file

Additional file 3 Test file for S2 split detection. Binary soundtrack of realistically simulated two S2 heart sound splits shown in Figure 6. Each split has its own characteristic interval. Click here for file

Additional file 4 Clinical Recording of VSD. Supplemental study that illustrates Heart Energy Signature (HES) characterization of Ventricular Septal Defect (VSD), illustrates accuracy of end point detection using phonocardiogram, various filters, HES. Click here for file

Additional file 5 Mitral Valve Prolapse with 3 clicks. sound track for Fig. 11. Click here for file

Additional file 6 Aortic Stenosis. sound track for Fig. 12. Click here for file

Additional file 7 Pulmonary Stenosis. sound track for Fig. 13. Click here for file

Additional file 8 Tetralogy of Fallot (acyanotic). sound track for Fig. 14. Click here for file

Additional file 9 Ventricular Septal Defect (VSD). Sound track for Additional file 4 Click here for file

## References

[B1] Mangione S, Nieman LZ (1997). Cardiac auscultatory skills in internal medicine and family practice trainees. JAMA.

[B2] Mangione S (1998). The teaching of cardiac auscultation during internal medicine and family medicine training-a nation wide comparison. Acad Med.

[B3] Roy DL, Sargent J, Gray J, Hoyt B, Allen M, Fleming M (2002). Helping family physicians improve their cardiac auscultatory skills with an inter-active CD Rom. J Contin Educ Health Prof.

[B4] Haney I, Ipp M, Feldman W, McCrindle BW (1999). Accuracy ofclinical assessment of heart murmurs by office based (general practice) pediatricians. Arch Dis Child.

[B5] Favrat B, Pecoud A, Jaussi A Teaching cardiac auscultation to trainees in internal medicine and family practice: does it work?. BMC Med Educ.

[B6] Fogel DH (1960). The innocent heat murmur in children; a clinical study of its incidence and characteristics. Am Heart J.

[B7] MacLaren MJ, Lachman AS, Pocock WA, Barlow JB (1980). Innocent murmurs and third heart sounds in black school children. Br Heart J.

[B8] U.S. Department of Health and Human Services, Center for Medicare and Medicaid Services (CMS). Obsolete and unreliable diagnostic tests. Medicare Coverage Issues Manual Sec 50-34 CMS Pub No 6 Baltimore, MD: CMS; effective January 1, 1997.

[B9] Baura GD (2005). The Business of innovation. MX: The Online Information Source for the Medical Device Industry.

[B10] Kassal J, Reeves W, Donnerstein R (1994). Polymer-based adhered differential output sensor for cardiac auscultation. Med Electronics.

[B11] McKusick VA, Webb GN, Humphries JN, Reid JA (1955). On cardiovascular sound: further observations by means of spectral phonocardiography. Circulation.

[B12] McKusick VA, Massengale ON, Wigod M, Webb GN (1956). Spectral phonocardiographic studies in congenital heart disease. Brit Heart J.

[B13] McKusick VA (1958). Cardiovascular sound in health and disease.

[B14] McKusick VA (1959). Spectral phonocardiography. Am J Cardiol.

[B15] Don Michael TA (1998). Auscultation of the Heart: A Cardiophonetic Approach. McGraw Hill.

[B16] Balster DA, Chan DP, Rowland DG, Allen HD (1997). Digital acoustic analysis of precordial innocent versus ventricular septal defect murmurs in children. Am J Cardiol.

[B17] Donnerstein R, Thomsen VV (1994). Hemodynamic and anatomic factors affecting the frequency content of Still's innocent murmur. Am J Cardiol.

[B18] Noponen AL, Lukkarinen S, Angela A, Sikio K, Sepponen R (2000). How to recognize innocent vibratory murmur. Comp in Cardiol.

[B19] Noponen AL (2005). 4^th ^Congress on Pediatric Cardiology. Poster Presentation.

[B20] Tovar-Corona B, Hind MD, Torry JN, Vincent R (2001). Effects of respiration on heart sounds using time-frequency analysis. Comp in Cardiol.

[B21] Tovar-Corona B, Torry JN (1998). Time-frequency representation of systolic murmurs using wavelets. Comput in Cardiol.

[B22] DeGroff CG, Bhatikar S, Herzburg J, Shandas R, Vlades-Cruz L, Mahajan RL (2001). Artificial neural network-based method of screening heart murmurs in children. Circulation.

[B23] Tavel ME, Katz H (2005). Usefulness of a new sound spectral averaging technique to distinguish an innocent systolic murmur from that of aortic stenosis. Am J Cardiol.

[B24] Kim D, Tavel ME (2003). Assessment of severity of aortic stenosis through time-frequency analysis of murmur. Chest.

[B25] Tavel ME (2006). Cardiac auscultation: a glorious past: and it does have a future. Circulation.

[B26] Tuchinda C, Thompson WR (2001). Cardiac auscultatory recording database: delivering heart sounds through the internet. Proc AMIA Symp.

[B27] Hayek CS, Thompson WR (2003). Wavelet processing of systolic murmurs to assist with clinical diagnosis of heart disease. Biomed Instrum Technol (Biomedical instrumentation & technology/Association for the Advancement of Medical Instrumentation).

[B28] Cohen L, Debnath L (2001). The uncertainty principle for the short time fourier transform and wavelet transform. Wavelet transforms and time frequency signal analysis.

[B29] Rein S, Reisslein M (2006). Identifying the classical music composition of an unknown performance with wavelet dispersion vector neural nets (extended version). Information Sciences.

[B30] (2002). Signal processing toolbox for use with Matlab, Version 5. Mathworks.

[B31] Qian S, Dapang CD (1996). Joint time-frequency analysis: method and applications. Prentice Hall, NJ.

[B32] Lang CW, Forinash K (1998). Time-frequency analysis with the continuos wavelet transform. Am J Phys.

[B33] Green JM, Wilcke JR, Abbott J, Rees LP (2006). Development and evaluation of methods for structured recording of heart murmur findings using SNOMED-CT post-coordination. J Am Med Inform Assoc.

[B34] Nigam V, Priemer R (2006). A dynamic method to estimate the time split between the A2 and P2 components of the S2 heart sound. Physiol Meas.

[B35] Nigam V, Priemer R (2005). Accessing heart dynamics to estimate durations of heart sounds. Physiol Meas.

[B36] Finley P, Warren AE, Sharratt GP, Amit M (2006). Assessing children's heart sounds at a distance with digital recordings. Pediatrics.

[B37] Marcus GM, Gerber IL, McKeown BH, Vessey JC, Jordan MV, Huddleston M, McCulloch CE, Foster E, Chatterjee K, Michaels AD (2005). Association between phonocardiographic third and fourth heart sounds and objective measures of left ventricular function. JAMA.

[B38] Collins SP, Lindsell CJ, Peacock WF, Hedger VD, Askew J, Eckert DC, Storrow AB (2006). The combined utility of an S3 heart sound and B-type natriuretic peptide levels in emergency department patients with dyspnea. J Card Fail.

[B39] Kudriavtsev VV, Kaelber D, Lazbin M, Polyshchuk VV, Roy DL New tool to identify Still's murmurs. Pediatric Academic Societies Annual Meeting.

[B40] Polyshchuk VV, Kudriavtsev VV Cardiovascular sound signature: method, process and format. USPTO Application, 2005; 0222515.

[B41] Polyshchuk V, Choy FK, Braun MJ (2002). New gear-fault-detection parameter by use of joint time-frequency distribution. AIAA J of Prop and Power.

[B42] Polyshchuk V (1999). Detection and quantification of the gear tooth damage from the vibration and acoustic signatures. PhD Dissertation.

[B43] Reed TR, Reed NE, Fritzson P (2004). Heart Sound analysis for symptom detection and computer-aided diagnosis. Sim Mod Practice and Theory.

[B44] de Bruijn NG (1973). A theory of generalized functions, with applications to Wigner distribution and Wyel correspondence. Nieuw Archief voor Wiskunde.

[B45] Wigner E (1932). On the quantum correction for thermodynamic equilibrium. Phys Rev.

[B46] Ville J (1948). Theorie et applications de la notion de signal analytique. Cables et Transmission.

[B47] Messiah A (1970). Quantum mechanics. translated by G.M. Temmer, North Holland, Amsterdam.

[B48] Cable CS, Ducharme NG, Hackett RP, Erb HN, Mitchell LM, Soderholm LV (2002). Sound signature for identification and quantification of upper airway disease in horses. Am J of Vet Res.

[B49] Cohen L (1995). Time frequency analysis. Prentice Hall, Englewood Cliffs, NJ.

[B50] Roy DL, Hoyt B Collection of heart sounds: EarsOn. CD Rom, CorSonics Corp, Halifax, NS.

[B51] Cohen L (1989). Time-frequency distributions – a review. Proceedings of the IEEE.

[B52] Mertins A (1999). Signal analysis. Wavelets, Filter banks, Time-frequency transforms and applications.

[B53] Claasen TACM, Mecklenbrauker VFG (1980). The Wigner distribution – a tool for time-frequency signal analysis, Part I: Continuous time signals. Philips J of Res.

[B54] Chen VC, Ling H (2003). Time frequency transforms for radar imaging and signal analysis. Artech House.

[B55] Bentley PM (1998). Time-frequency and time-scale techniques for the classification of native and bioprosthetic heart valve sounds. IEEE Trans Biomed Eng.

[B56] (2006). Bsignal – Heart energy signature visualization system, Version 3.5. Product manual.

[B57] Wigner quasi-probability distribution. http://en.wikipedia.org/wiki/Wigner_quasi-probability_distribution.

[B58] Braunwald E, Antman EM, Beasley JW (2002). ACC/AHA guideline update for the management of patients with unstable angina and non-ST-segment elevation myocardial infarction -2002: summary article: a report of the American College of Cardiology/American Heart Association Task Force on Practice Guidelines (Committee on the Management of Patients With Unstable Angina). Circulation.

[B59] Freeman AR, Levine SA (1933). The clinical significance of the systolic murmur: A study of 1,000 consecutive "non-cardiac" cases. Ann Intern Med.

[B60] Nygaard H, Thuesen L, Terp K, Hasenkam JM, Paulsens PK (1993). Assessing the severity of aortic valve Stenosis by spectral analysis of cardiac murmurs (spectral vibrography). Part II: Clinical Aspects. J Heart Valve Dis.

[B61] Kvart C, French AT, Fuentes VL, Haggstorm J, McEwan JD, Schober KE (1998). Analysis of murmur intensity, duration and frequency components in dogs with aortic stenosis. J Small Anim Pract.

[B62] Bulgrin JR, Rubal BJ, Thompson CR, Moody JM (1993). Comparison of short-time Fourier, wavelet and time-domain analyses of intracardiac sounds. Biomed Sci Instrum.

[B63] Rabin A (1967). Auscultation of the Heart.

