# Supplementary material for: Heart energy signature spectrogram for cardiovascular diagnosis
Source: Biomed Eng Online. 2007 May 4;6:16. doi: 10.1186/1475-925X-6-16 (PMC1899182; doi:10.1186/1475-925X-6-16)
Supplement: Additional file 4 — Clinical Recording of VSD. Supplemental study that illustrates Heart Energy Signature (HES) characterization of Ventricular Septal Defect (VSD), illustrates accuracy of end point detection using phonocardiogram, various filters, HES. [file 1475-925X-6-16-S4.pdf]

# Heart energy signature spectrogram for cardiovascular diagnosis

**Biomedical Engineering Online, 2007**

**Vladimir Kudriavtsev, Vladimir Polyshchuk, Douglas L. Roy**

## HES-based characterization of recording of a small VSD

**Objective: to demonstrate that HES can help to quantify heart sound data, and that it is capable of estimating heart sounds durations, endpoints, intensity and frequency variation, and S2 split. In parallel, ambiguities of end point estimations using PCG only, and PCG ambiguities due to filtration, are demonstrated. Direct comparison with STFT spectrograms is provided. First two beats of the heart sound are presented in Additional file 9.**

### 1. HES endpoint detection

**Fig.1 is four heart beats of a recording of a patient with a small VSD. A Welch-Allyn (Meditron) digital stethoscope and computer software of our design were used to record.**

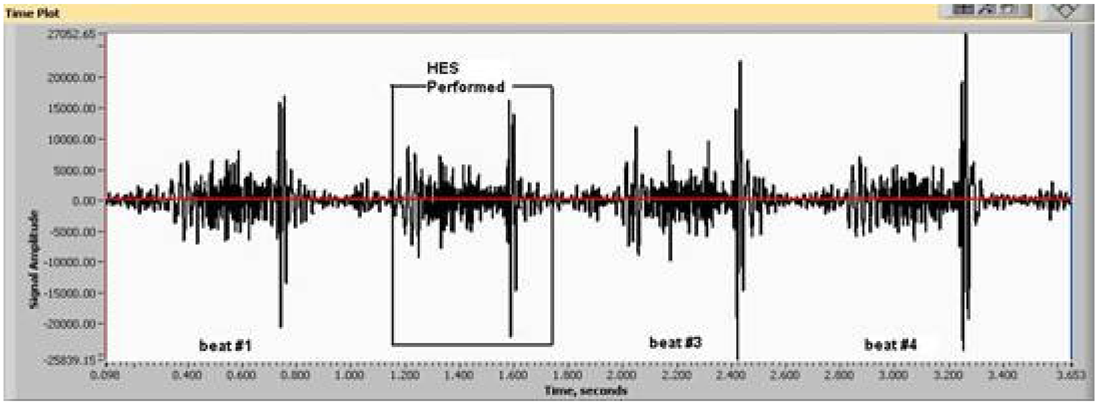


**Figure 1. Phonocardiogram showing 4 VSD heart beats**

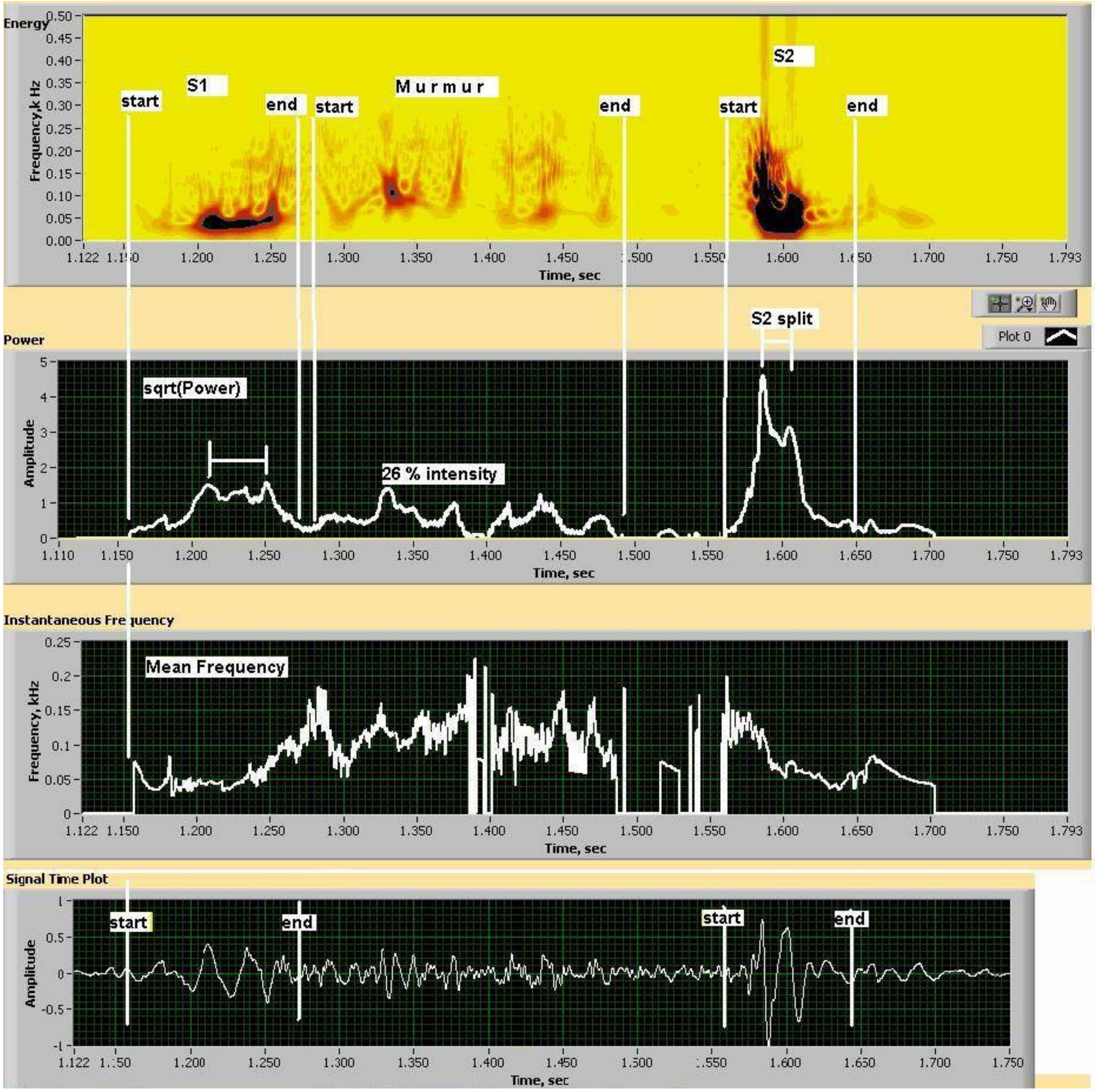


**Figure 2. Heart Energy Signature Format , part 1, showing HES of heart beat #2 marked on Figure 1**

Generally exact start and end points of the sound waveforms (S1,S2 and murmur) on the PCG (signal time plot) are not immediately obvious, which is also the case for the recording shown on Figures 1 and 2. However, examination of HES Power plot *and* analysis of instantaneous frequency plot (both are extracted from HES) allows precise *estimation of* end points.(as marked on Fig 2). Close S1 split is present on the power plot (two small peaks) and narrow S2 split can also beseen. S2 split can be accurately measured as time distance between S2 power plot peaks. (Figure 2).

End points estimated using HES are marked on Figure1. This allows measurement of S1, S2 and murmur time durations (see also Table 1) below.

Instantaneous frequency variation is demonstrated and quantitatively measured. For example murmur frequency does not exceed 220Hz, with time average frequency being much lower

(125Hz) and multiple peaks within 150 – 180 Hz. range. Murmur frequency is an important measure indicating abnormality, innocent murmurs being usually below 110 Hz. HES Power plot here is presented in <square root of power> format and allows estimation of murmur energy peak (at time moment 1.33 sec) as 26% of the maximum S2 sound power plot, in keeping with a murmur of grade 2/6 intensity.

**VSD Frequencies**

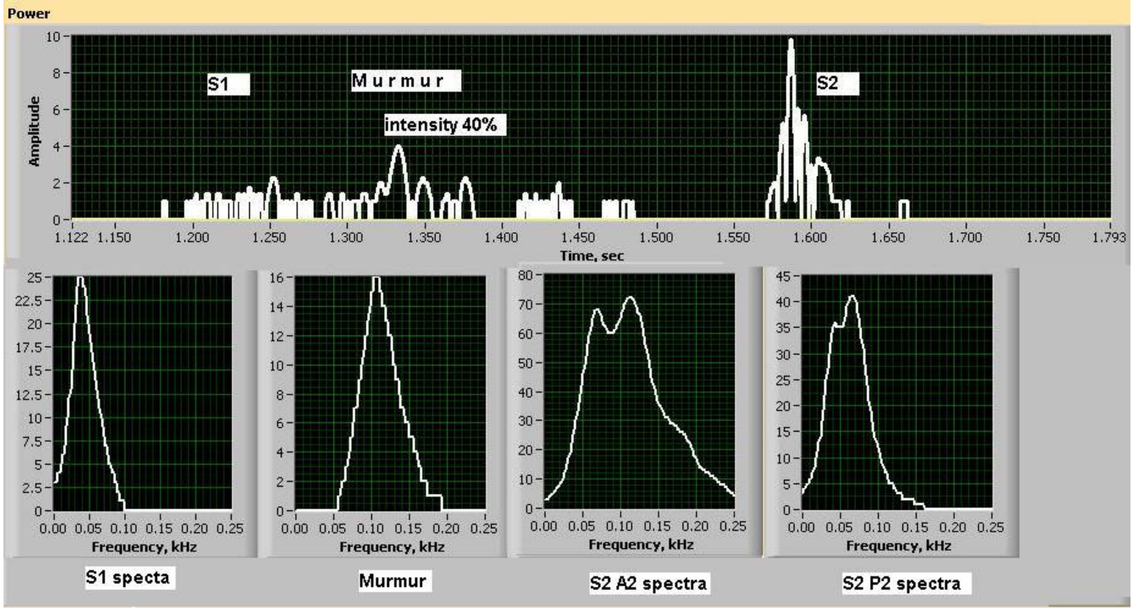


**Fig 3. Continuation plot showing other details of HES format – instantaneous power distribution for a given frequency and several instantaneous frequency distributions for Time instances associated with S1, murmur and S2 (A2 and P2).**

Instantaneous Power plot (extracted at the lead frequency 115Hz of murmur peak (1.33 sec) energy -115Hz) shows power intensity at this frequency to be 40% of S2 maximum.

Four instantaneous frequency spectra plots are shown and all are extracted along the vertical lines from the HES image (Figure 2) at different time instances. Spectral spread is measured as spectral width at half of spectral intensity. S1 (1.23 sec) frequency spectra peaks at 40Hz and has 35 Hz spread. Murmur (at 1.33 sec) has peak frequency of 110Hz and 55Hz spread. S2 frequency for A2 moment (first peak on the power plot) is 120 Hz and spread is 90 Hz. P2 peak is 70HZ, with half intensity spread being 60Hz.

**2. Illustrating endpoints detection using PCG, filters and HES**

**A) S2 split estimation from PCG with two filters**

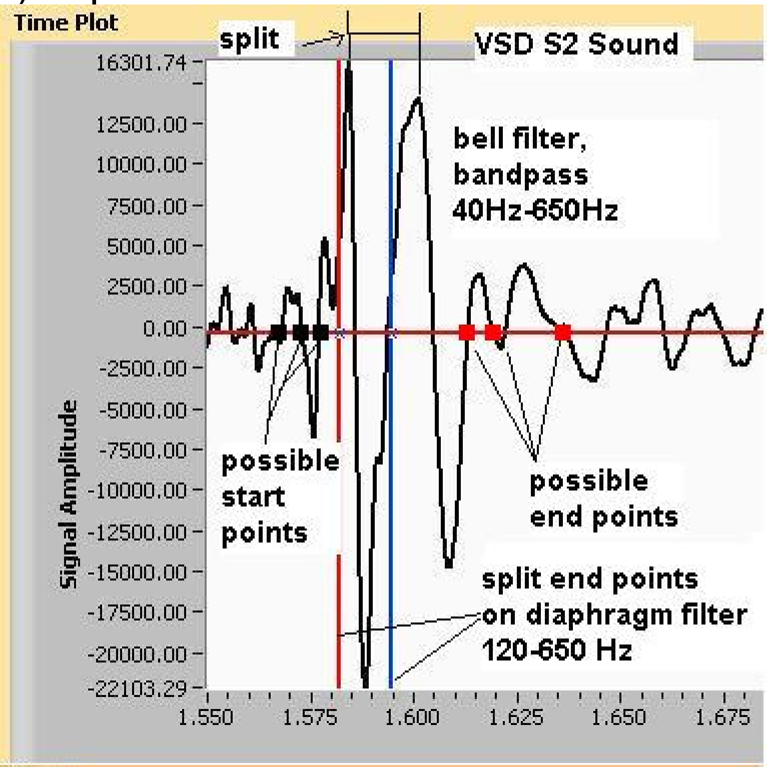


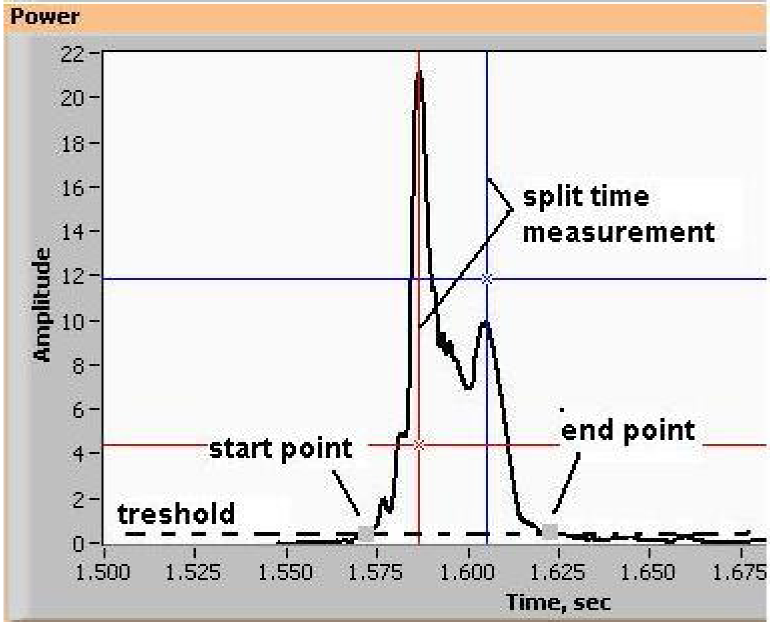


**B) HES Power Plot of S2 (second beat of Figure 1)**

**Figures 4A and B. S2 Split Estimation**

S2 split measurements are illustrated in Figures 4 A and 4B. On Figure 4A we mark with squares potential locations of end and start points for S2 as there is some ambiguity where S2 waveform may actually start and end. End points for A2 and P2 (S2 split) are marked with vertical lines. Waveform shown on Figure 4A was bell filtered and alternative location of split end points are

marked for diaphragm filtered waveform. Considerable discrepancy in split estimation between the two widely acceptable filters are seen. Summary of several split estimations is presented in Table 1.

Possible end points are marked as squares on the plot. The correspondingly S2 duration is different however, demonstrating ambiguity of PCG analysis.

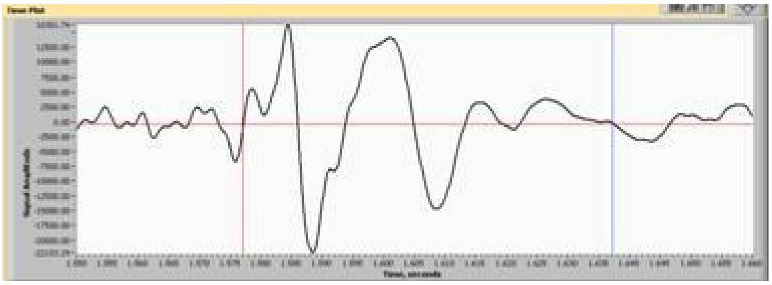

**C) Bell filter –**

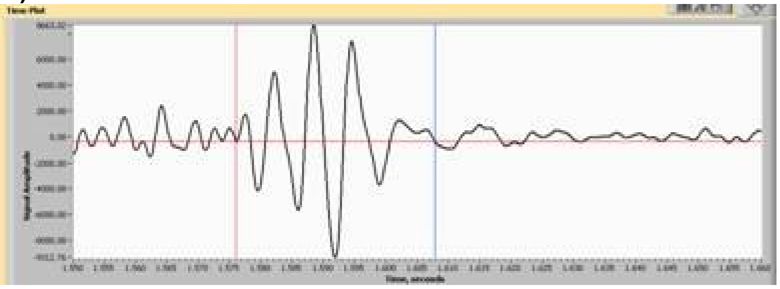

**D) Diaphragm filter with end points shown-same**

**Figure 4C and D. S2 signal plots obtained using two different filters (horizontal time scale is unchanged, start and endpoints are marked with vertical lines)**

S2 duration and S2 split duration is different depending on which filter is used. This is of fundamental importance. The number of peaks and peak visual depiction are obviously different.

HES power plot (Figure 4B) shows no ambiguity of start and end points and split duration. Split is determined as distance between peaks on power plot (figs.2 and 4B) and S2 start and end points are determined at the intersection with the horizontal cut-off threshold line. It is reasonable to set threshold at value equal 2.5% of peak power.

Murmur start and end points were also measured from the PCG and Power plot (Fig. 2). PCG measurements suggest murmur length = 0.298 sec (96% systolic duration). HES power plot suggests murmur duration of 0.2 sec (66% systolic duration). Here systolic duration (distance between end of S1 and beginning of S2 is equal to 0.306 sec). HES is more accurate in this case as it objectively tracks power that goes into the sound.

**3. Filter dependent signal proportions**

Amplitude ratios of PCG plot do change with change in filtration scheme. Murmur intensity (for this recording) changes very little. It is estimated as 33% for bell filter and as 36% for diaphragm filter. S1/S2 ratio changes considerably being 17% for the diaphragm filter and 15% for bell filter.

**Table 1. S2 split and S2 duration estimation (from the PCG-manual)**

| | S2 length | S2split | Comment |
|---|---|---|---|
| **Diaph filter** | 0.0315 | 0.013 | (underestimate) |
| **Bell filter** | 0.06 or 0.04 | 0.017 | (overestimate) |
| **HES Power** | 0.047 | 0.019 | (in between, which is logical) |
| **STFT Image** (approximately) | 0.33 | unable to estimate | |

**STFT Spectrogram Time Duration Characteristics**

The same heart sound recording was analyzed using Meditron Analyzer software, using default options (Hanning STFT window). Window size was picked to be equal to 1024 data points. Results are shown in Figure 5 where for comparison purpose we mark end points obtained from the HES and PCG plots against the best possible estimate of endpoints from this STFT spectrogram.

Used window size presents reasonable compromise between the time and frequency resolution and was selected to properly represent frequencies that were estimated from HES. For example this plot shows S2 A2 frequency in the range up to 200Hz and murmur frequency close to 100Hz. This is approximately comparable with HES image results, but STFT has considerable time smear and it is impossible to separate S1 from S2 and to determine end points accurately. The best graphical estimate of S2 duration is 0.33 sec for STFT (Figure 5) vs. 0.047 for HES (Figure 2) **vs. 0.06 sec for PCG (Figure 1). Also S2 s**plit is not clearly visible as on HES image. The best estimate of STFT A2 location is marked on Figure 5 and is shifted forward. S2 start is also shifted forward and S2 end is shifted backwards.

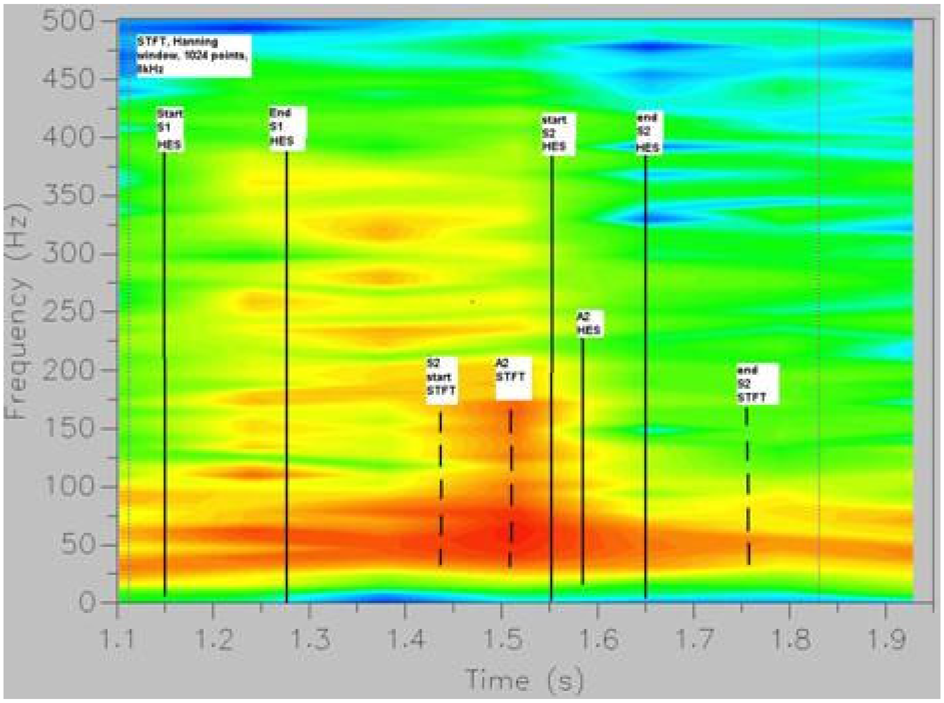


**Figure 5. STFT Spectrogram of the second heart beat shown on Figure 1.**

### 4. Comparison of STFT spectrogram obtained for various window sizes with HES spectrogram

Figure 6 demonstrates the visual comparison of time-frequency resolution of STFT spectrogram with HES spectrogram. Short STFT window provides better time resolution at the expense of frequency resolution. Two heart beats are shown which directly correspond with first two heart beats shown on Figure 1. All four pictures use same time and frequency scale, with maximum frequency being 500Hz. Spectrogram image of 1024 resolution is also shown on Figure 5. Ambiguity of STFT spectrogram is clearly demonstrated. For example, S2 A2 peak frequency estimate of the second heart beat varies between 230Hz. to 95 Hz. and has uncertainty (for 2048) between 25 to 325 Hz due to artifacts. Same peaks are estimated at 250Hz from the HES image and sharp contrast of HES image resolution with STFT spectrogram is obvious. HES shows complex murmur structure that contain several time splashes of energy. Corresponding HES frequency characteristics are shown in detail on Figures 2A and 2B.

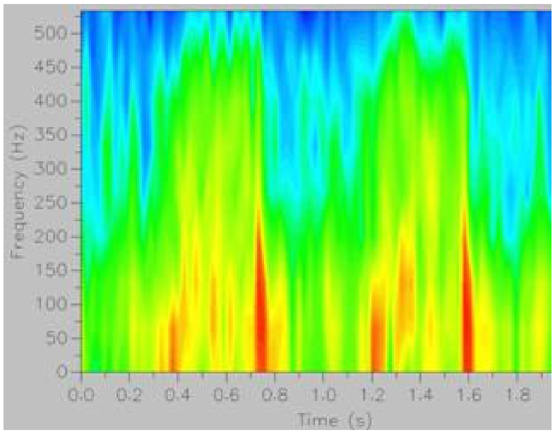


**Window w=128 points**, S2 (1.6 sec) peak frequency 230 Hz. No low frequency resolution, good time resolution

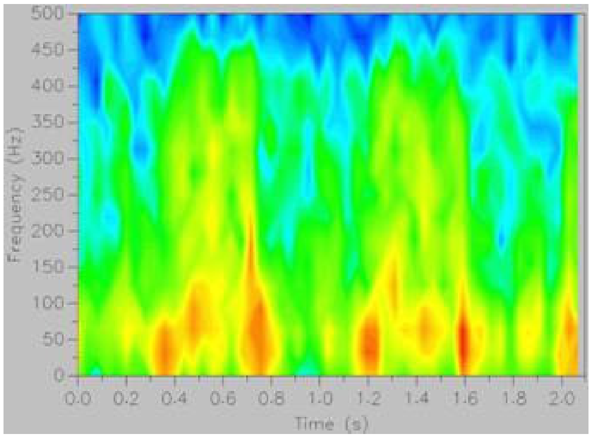


**Window w=256 points**, S2 (1.6 sec) peak frequency 110Hz, compromise: frequency semi accurate

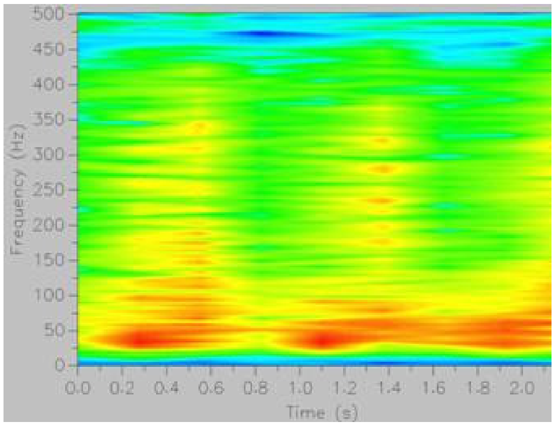


**Window w=2054** S2 (1.4 sec) peak frequency 95Hz, artifacts up to 325Hz.

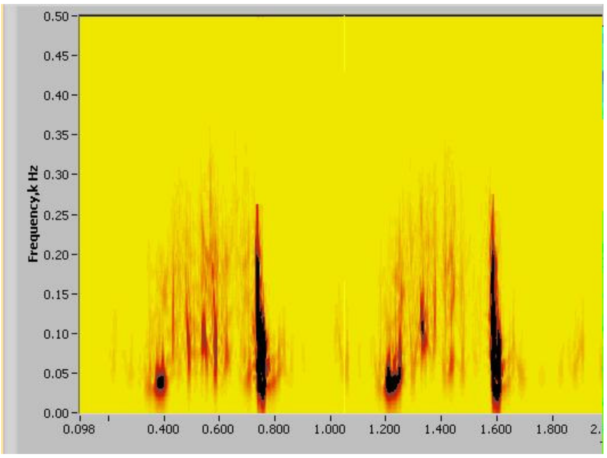


**HES**: peak frequency at 250 Hz, clear resolution

**Figure 6. Visual Comparison of STFT spectrogram with the HES spectrogram**
